# Stereodiscrimination of guests in chiral organosilica aerogels studied by ESR spectroscopy

**DOI:** 10.3762/bjnano.16.140

**Published:** 2025-11-13

**Authors:** Sebastian Polarz, Yasar Krysiak, Martin Wessig, Florian Kuhlmann

**Affiliations:** 1 Institute of Inorganic Chemistry, Leibniz-University Hannover, Callinstrasse 9, 30167 Hannover, Germany

**Keywords:** chirality, confinement chemistry, ESR spectroscopy, organic inorganic hybrids, porous materials

## Abstract

Macroporous materials containing surfaces with chiral groups are highly relevant for applications in the chromatographic separation of enantiomers. Despite these materials being highly engineered and commercially available, optimization was often done empirically. A rational design of future and improved solid phases for chiral chromatography requires that one understands how the chemical structure of a surface influences the stereoselectivity of the enantiomers at the surface. Despite the difference in the interaction enthalpies being only in the 1–2 kJ·mol^−1^ range, an ideal surface would exclusively interact with one enantiomer. However, the question of which selectivity is sufficient or necessary to reach separation is an important point. We have employed the two enantiomers of a chiral, nitroxide-based spin probe as guests in organo-modified macroporous host materials and applied ESR spectroscopy as a tool to investigate their rotational mobility. Using a well-established and commercially available material confirmed the method’s reliability. The data underline how crucial the choice of the right solvent is if one wants to reach sufficient selectivity. Together with a series of custom-made organosilica aerogels, it is shown that adjusting solvent and surface properties so that the two enantiomers (+) and (−) experience a different chemical environment is key. Otherwise, there might be a dynamic equilibrium between surface-adsorbed and mobile spin probes without stereodifferentiation. With this knowledge, it was possible to reach higher selectivity values than for the commercial material. A particularly interesting result was that better performance could be achieved if one attaches bulky, hydrophobic groups directly to the stereocenter. The effect of such neighboring groups on the enantioselectivity highly depends on the distance they have to the stereocenter.

## Introduction

Chiral materials represent an evolving research field that focuses on materials whose structures lack mirror symmetry [1–3]. The materials exhibit chirality, and a good overview of important developments was given by Kotov and coworkers [4]. Chiral optical materials have unique optical activity, displaying phenomena such as circular dichroism and optical rotation. These characteristics are harnessed in applications like sensors, optical devices, and polarized materials. Material chirality can also lead to unusual electromagnetic properties, enabling the development of metamaterials with negative refractive indices or chiral photonic crystals, which can manipulate light in novel ways. Another exciting area of research in chiral materials is developing supports for enantioselective catalysis [5–6]. Enantiomers are stereoisomers that are non-superimposable mirror images of each other. Enantioselectivity refers to the preference of a chemical reaction to produce one enantiomer over another. Stereoselectivity is a broader term that includes both enantioselectivity and diastereoselectivity. Diastereomers contain more than one stereocenter and are not mirror images of each other. In a diastereoselective reaction, there is a preference for forming specific configurations at multiple stereocenters. The formation of enantiomers is typically the result of a diastereomeric couple at a crucial point of the process. Thus, chiral materials are crucial in asymmetric synthesis, where the selective production of a desired enantiomer is needed, particularly in pharmaceuticals [7]. Chiral metal-organic frameworks [8] and nanoporous materials [9–10] are emerging as vital components in enantioselective catalysis due to their high surface area and customizable chiral environments.

A highly important and highly developed area of application of chiral materials possessing a high surface area is chromatography [11–13]. Techniques like high-performance liquid chromatography (HPLC) [13] and gas chromatography [14] are adapted for chiral separations, providing robust, reproducible methods for enantiomeric resolution. Chiral chromatography is a pivotal technique in the separation and analysis of chiral compounds, particularly in the pharmaceutical industry, where the distinction between different enantiomers of a compound can be critical [12]. Enantiomers often exhibit vastly different biological activities; one may be therapeutically beneficial, while the other may be inactive or even harmful. Thus, the pharmaceutical industry heavily relies on chiral chromatography to ensure the efficacy and safety of chiral drugs. Beyond pharmaceuticals, chiral chromatography is also essential in agrochemicals, flavors, fragrances, and many other chemicals whose stereochemistry plays a role in their biological interactions and sensory properties.

The essence of chiral chromatography involves using chiral stationary phases or derivatizing agents, enabling the separation of enantiomers based on their interactions with these chiral entities. It is obvious that the transport properties of the two enantiomers must be different for them to be separated successfully. Transport in porous media is a topic with much broader and more general relevance than just for chromatography. Transport processes in porous materials are fundamental phenomena that influence the behavior and performance of a wide range of natural and engineered systems. They are relevant in applications including catalysis, filtration, energy storage, environmental remediation, and construction. The key transport processes in porous materials include diffusion, advection, capillary action, and sometimes reactions that might occur within the pores, highly complex phenomena one tries to understand as detailed as possible [15–17]. Laemmerhofer gave an extensive description of the enormous progress of liquid chromatography with chiral, stationary phases made over several decades [18]. The author analyzes that a molecular understanding of the recognition mechanisms on chiral surfaces is still scarce, particularly regarding dynamic aspects. Meanwhile, theoretical methods have helped to gain deeper insights, but there is still a gap in experimental data.

Different experimental methods are used to investigate transport in porous media. Chromatography itself has to be counted as one method that delivers information about the macroscopic properties of a material. In essence, one sees here the interplay of all processes contributing to transport. Gathering information about the phenomena at short(er) distances and short(er) timescales and about the correlation between the mobility of guest species and intermolecular interactions with functionalized surfaces has proven challenging. For the separation of the individual factors, researchers developed powerful nuclear magnetic resonance (NMR) techniques. Great success was reached using pulsed field gradient (PFG)-NMR [19–21]. PFG-NMR incorporates the application of magnetic field gradients in addition to the uniform external magnetic field. A sequence of gradient pulses is used to label the position of the nuclear spins. The first pulse encodes spatial information; if the spins move (diffuse) during the interval, a second gradient pulse will partially or fully refocus them. The extent to which the spins can be refocused depends on their diffusional displacement during the interval between the gradient pulses. Thus, PFG-NMR is sensitive for processes occurring on length scales from ca. 10 nm to several millimeters and at the milliseconds-to-seconds timescale. Besides the fact that NMR is an established technique, an obvious advantage is that countless compounds contain NMR-active nuclei, and the interested reader is referred to one of the excellent review articles by Kärger and coworkers [21–23]. However, the solvents and the matrix of the porous host can also be NMR-active, which makes measurements more difficult.

There are other valuable methods for investigating transport in porous media such as gas-adsorption methods, X-ray tomography [24–25], neutron imaging techniques [26–27], optical imaging techniques [27–28], or impedance spectroscopy [29–30]. For all of them, it is difficult to access the sub-10 nm domain and to obtain information about the dynamics of guests confined to the porous system on short timescales below milliseconds. Here, electron spin resonance (ESR) has some advantages. Because of the temporal dimension of these rotational dynamics at the microseconds timescale, one is able to gather information about transport processes happening at 1–3 nm. If one selects an appropriate spin probe, the ESR spectra contain much valuable information. Line shape analysis and spin relaxation times can provide information on the motion and reactivity of trapped radicals. This can reveal interactions between the guest species and the pore walls or other guest molecules, helping to elucidate confinement effects.

Organic nitroxides [31–32] have proven to be of extreme value for advanced ESR investigations [33–35]. Nitroxide spin probes are highly stable due to steric shielding by the methyl groups. It was shown that the line shape of the characteristic ESR signals of nitroxide spin probes depends strongly on molecular dynamics. Because of the anisotropy of Zeeman and hyperfine coupling, one can extract detailed information about the rotational dynamics. Highly mobile spin probes with free rotation are characterized by a spectrum with three narrow lines [36–37]. Anisotropic broadening of the spectrum is indicative for retardation of rotation due to additional intermolecular interactions. The hyperfine coupling of the electron spin with the nuclear spin of ^15^N provides detailed information about the local environment. Furthermore, dipolar coupling to other electron spins in the direct environment allows for the determination of distances from 0 to 10 nm [38–39]. Giamello et al. have discussed in detail how this effect can be used to characterize the interaction of spin probes with surfaces [40]. Mastai and coworkers have used ESR spectroscopy to probe the chirality of crystals [41].

Our group used ESR to investigate the rotational dynamics of paramagnetic spin probes in functionalized, mesoporous organosilica materials [42–46]. Using 3-carboxypropyl (3CP) as a spin probe, we observed that the ESR spectra consist of two species, one rotating freely and the other one much more slowly with tumbling dynamics [42]. Because these two species can be correlated to 3CP in the solvent (fast rotation) and 3CP adsorbed at the surfaces (slow dynamics), quantitative evaluation of the spectra allowed for investigating various confinement effects. Our focus was on understanding how a particular functionalization of the surfaces in combination with 3CP and the solvent influences transport properties, and novel ESR imaging techniques were used to determine diffusion coefficients [44]. In the mentioned studies, we have always used racemic 3CP containing both enantiomers. Because of our previous work on mesoporous organosilica materials containing chiral groups [47–48], particularly amino acid derivatives [49], we became interested in observing the two enantiomers (+)-3CP and (−)-3CP separately [45]. Because of the small pores (<10 nm), the confinement situation was very strong and diffusion was limited. The first indications reported were that materials with larger pores have promising properties, too.

The paper here focuses exclusively on aerogel materials made of amino acid-functionalized organosilica. It is organized as follows: First, we present a set of materials that differ in their chemical architecture. We then use ESR spectroscopy for investigating the dynamics of (+)-3CP and (−)-3CP for determining stereodiscrimination. The situation will be compared to a commercial chiral chromatography material. Finally, we try to explain the observations by a more detailed picture of the local environment of the spin probe.

## Results and Discussion

### Achiral materials

**Figure 1:**
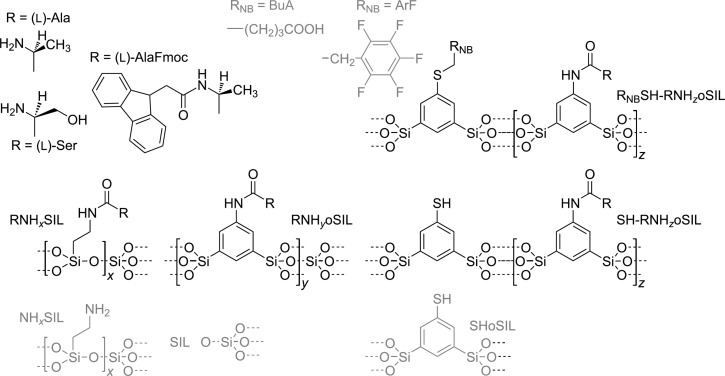
Genealogy of the Materials used in the current study. Achiral materials as references (grey); materials containing chiral selectors (black).

Three achiral aerogel materials (containing no chiral centers) were prepared as reference systems (Figure 1, grey structures). One material is pure silica, referred to as SIL; the second material, named NH*_x_*SIL, contains amino groups (NH_2_), and the third is composed of thiophenyl-containing organosilica [HSC_6_H_3_(SiO_1.5_)_2_], referred to as SHoSIL.

SILs are well known in the literature and were prepared accordingly [50]. For similar hydrolysis and condensation kinetics compared to the organosilane sol–gel precursors (see below), tetraisopropoxysilane (Si(OiPr)_4_) was used as a sol–gel precursor. After solvent exchange of the wet gel, supercritical drying with carbon dioxide delivered the aerogel. Characterization data for the prepared SIL is shown in Supporting Information File 1, Figure S1. A monolithic material with a large porosity and a polydisperse pore system in the size range *D*_pore_ = 50–300 nm was obtained. The specific surface area is *A*_BET_ = 946 m^2^·g^−1^ according to nitrogen physisorption measurements.

NH*_x_*SIL was prepared by co-condensation of tetraalkyloxysilanes (e.g., Si(OMe)_4_) with a second sol–gel precursor such as aminopropyltriethoxysilane (APTMS, (MeO)_3_SiPrNH_2_). One can control the amine content, *x*, by adjusting the ratio of the two precursors in the precursor sol. A material possessing 10% amine groups will be denoted NH*_x_*_=0.1_SIL (see the Experimental section and Supporting Information File 1, Figure S2).

Silsesquioxane compounds of the type R-Ph(Si(OiPr)_3_)_2_ with R = –COOH, –NH_2_, –N_3_, etc. have been used in our group for a long time to prepare various porous organosilica materials [51–52], also including aerogels [53–54]. The thiol precursor HS-Ph(Si(OiPr)_3_)_2_ was established in 2014 for making periodically ordered mesoporous organosilica [55–56], which are characterized by pore sizes in the sub-10 nm range. Because large pore sizes are beneficial for mass transport, and macro-to-mesoporous materials are more frequently used for chromatography [57], we concentrate on aerogels in our current study. An aerogel prepared from 1,3-bis(triisopropoxysilyl)thiophenol is presented here for the first time. The relevant characterization data are shown in Supporting Information File 1, Figure S3.

### Chiral materials

A selection of different materials containing amino acids as chiral selectors was prepared (Figure 1). The advantages of using amino acids as the chiral constituent are that they are highly available from the natural pool and their coupling chemistry is highly developed. For instance, serine (Ser) or alanine (Ala) can be attached to NH*_x_*SIL via amid bonds using a fluorenylmethyloxycarbonyl (Fmoc)-protected amino acid, followed by detachment of the protection group (see Experimental section and Supporting Information File 1, Figure S4).

**Scheme 1:**
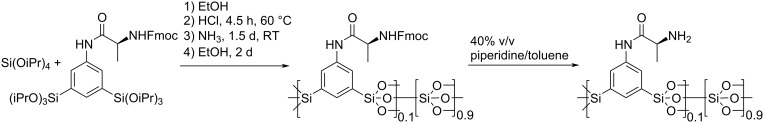
Synthesis route to AlaNH_10_oSil.

Alternatively, one can prepare chiral oSILs by co-condensation of Si(OiPr)_4_ with a sol–gel precursor that already contains the amino acid. An example is an Ala-modified 1,3-bis(triisopropoxysilyl)aniline [45,49] as shown in Scheme 1.

**Figure 2:**
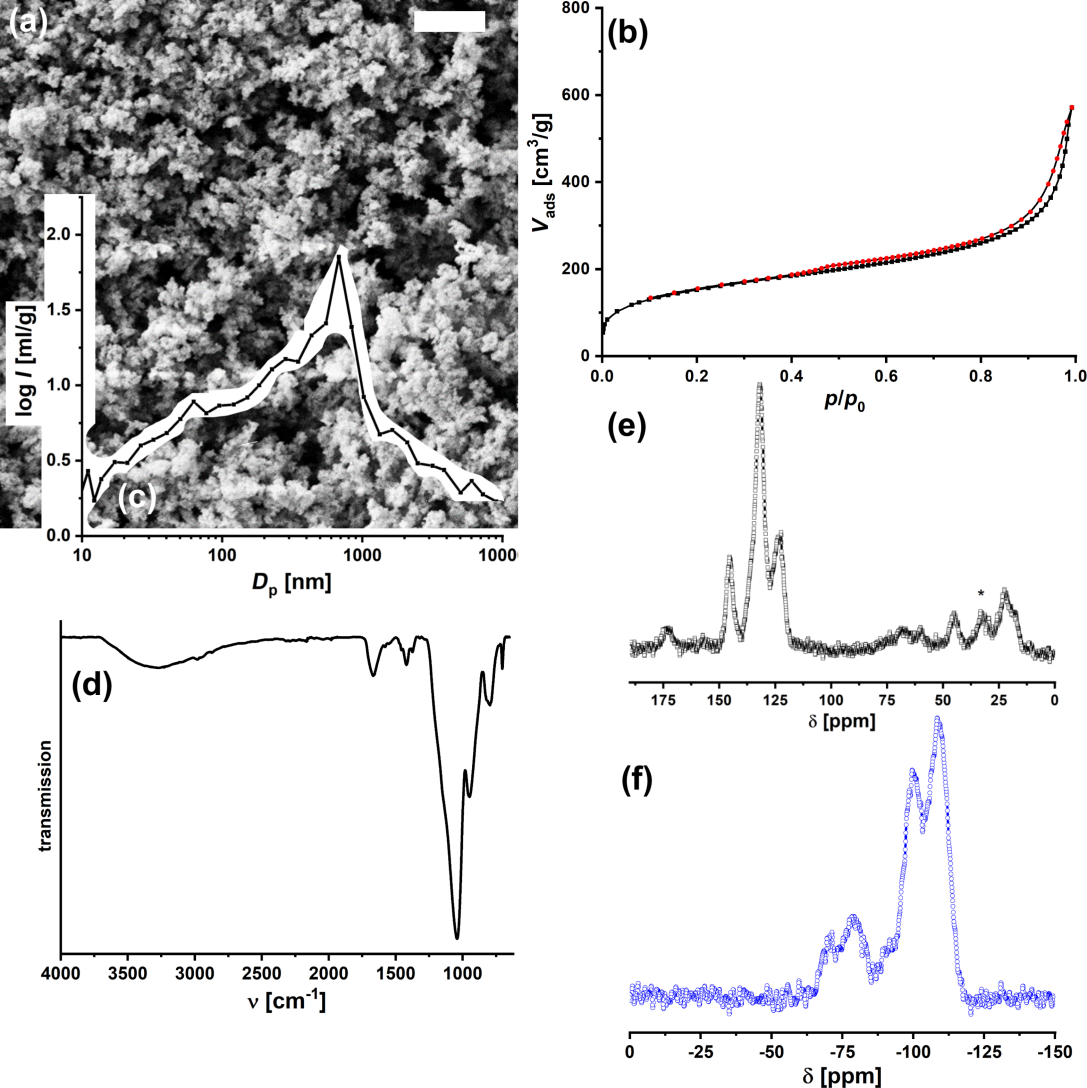
Characterization of AlaNH_10_oSIL. (a) SEM micrograph (scale bar = 1 μm). (b) N_2_ physisorption isotherm. (c) Pore-size distribution obtained from Hg intrusion porosimetry. (d) FTIR (ATR) spectrum. ^13^C (e, black; * = spinning side band) and ^29^Si (f, blue) MAS NMR spectra.

The resulting material was characterized by scanning electron microscopy (SEM; Figure 2a); it displays the typical aerogel structure with a random pore system and polydisperse sizes from 30 to 250 nm. The high porosity was confirmed by nitrogen physisorption analysis (Figure 2b). The isotherm is typical for a material with large mesopores and macropores, and the specific surface area, *A*_BET_, is 552 m^2^·g^−1^. The meso-macroporous structure was also found by mercury intrusion porosimetry (Figure 2c). The FTIR spectrum is consistent with the proposed composition (Figure 2d). The vibrations at ν = 3000–3600 cm^−1^ are characteristic for NH and OH groups. At 2982 cm^−1^, one sees the CH groups. Amide bands are seen at 1666 cm^−1^ (amide I) and 1557 cm^−1^ (amide II), and the bands at 1046 and 948 cm^−1^ originate from the siloxane network (Si–O–Si). The ^13^C magic angle spinning (MAS) nuclear magnetic resonance (NMR) spectrum (Figure 2e) displays signals for Ala-CH_3_ (δ = 16.0 ppm), Ala-CH (45.3 ppm), aromatic C (123.3, 132.1, and 145.1 ppm), and amide C=O (173.6 ppm).

We have also tested the possibility of increasing the amino acid content by using more of the phenyl-bridged sol–gel precursor. An aerogel material, AlaNH_15_oSil, could be prepared successfully (see Supporting Information File 1, Figure S5). A further increase of the chiral selector was not possible as AlaNH_25_SIL is no longer an aerogel material but is composed of large agglomerated colloids (see Supporting Information File 1, Figure S6). SerNH_10_oSil was prepared analogously using a serine-modified sol–gel precursor; the data is given in Supporting Information File 1, Figure S7.

**Scheme 2:**
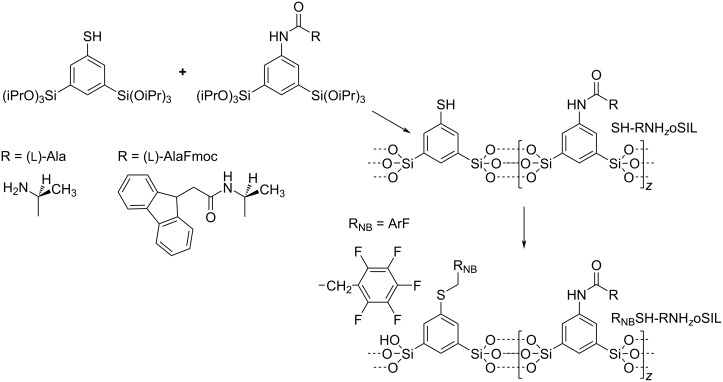
Synthesis route towards organosilica aerogels containing neighboring groups and chiral functionalities.

The alanine-modified precursor can also be used for co-condensation with the thiol precursor as shown in Scheme 2. The co-condensation of the latter precursor with the aforementioned Fmoc-protected, alanine-modified precursor delivers SH-FmocAlaNH_z_oSIL aerogels. After deprotection, one obtains SH-AlaNH_z_oSIL; the data is given in Supporting Information File 1, Figure S8. In particular, energy-dispersive X-ray spectroscopy analysis proves the presence of sulfur and, thus, the successful incorporation of the thiophenyl groups.

The value of the thiol groups is that almost any functional group can be attached by the so-called click chemistry or, in this case, thiol-Michael addition [58–62]. The methodology can be used for further functionalization of the aerogel materials [63]. For instance, the addition of perfluorostyrene affords ArFSH-AlaNH_10_oSil and, thus, a material containing a hydrophobic environment around the stereocenter. Characterization data are shown in Supporting Information File 1, Figure S9.

### Chiral spin probes in achiral hosts

The enantiomerically pure nitroxides are not commercially available. Therefore, 3CP was prepared according to [64], and the racemate was separated by fractional crystallization using (*S*)-(−)-1-phenylethylamine for (+)-3CP and (*R*)-(+)-1-phenylethylamine for (−)-3CP as indicated in Supporting Information File 1, Figure S10. The enantiomeric purity was checked after each crystallization step using the reported specific rotation value of 79° [65]. Enantiomeric excess values (ee) of 97% for (+)-3CP and 95% for (−)-3CP were obtained.

**Figure 3:**
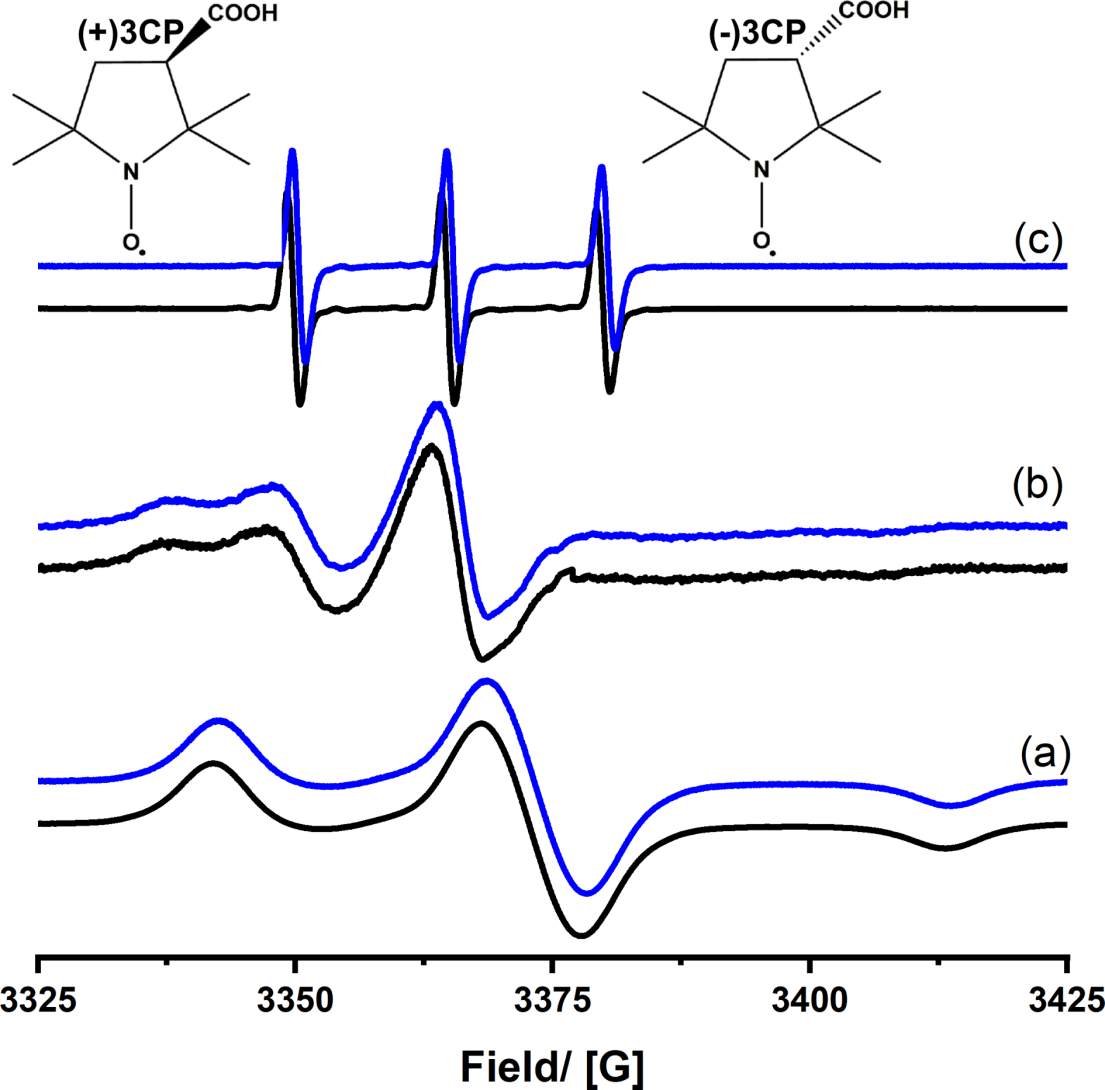
cw-ESR spectra of (+)-3CP (black) and (−)-3CP (blue) dissolved in ethanol and infiltrated into achiral silica aerogels recorded at different temperatures: (a) *T* = 103 K, (b) *T* = 183 K, and (c) *T* = 273 K.

The 3CP probe can be infiltrated into different hosts, and, from continuous wave (cw) ESR spectra, various information about the molecular dynamics can be obtained, as we have also discussed in several of our past papers [42–46]. Exemplarily, Figure 3 shows the behavior in an achiral silica aerogel. Because of the large pore size, the spin probe behaves almost like in a free solution. The spectra recorded at *T* = 273 K are characteristic of a freely rotating 3CP molecule. *T* = 103 K is below the melting point of ethanol. Therefore, one obtains a solid-state spectrum of 3CP. At *T* = 183 K, one can see that the spectrum contains both characteristics, some freely rotating 3CP and some molecules with slower, tumbling dynamics. As shown in the past, one can assign the slow component to spin probe molecules that are located near the surface of the porous host and, thus, are likely to be adsorbed to the surface. Fitting the spectra quantitatively yields the ratio between fast (non-adsorbed) and slow (adsorbed) species. For silica aerogels, one can see that, only at low temperatures, there is a notable fraction of surface-adsorbed species, which indicates that there is weak confinement and weak interaction between the pore walls and 3CP. The data recorded at room temperature shows no indication of surface-adsorbed compounds, and only quickly rotating 3CP is seen. If one compares the spectra obtained for the two enantiomers of 3CP, there is no difference (Figure 3). This result is expected because, for a porous host containing no chiral groups, there should be no difference in dynamics of the two compounds.

### Chiral spin probes in chiral hosts

**Figure 4:**
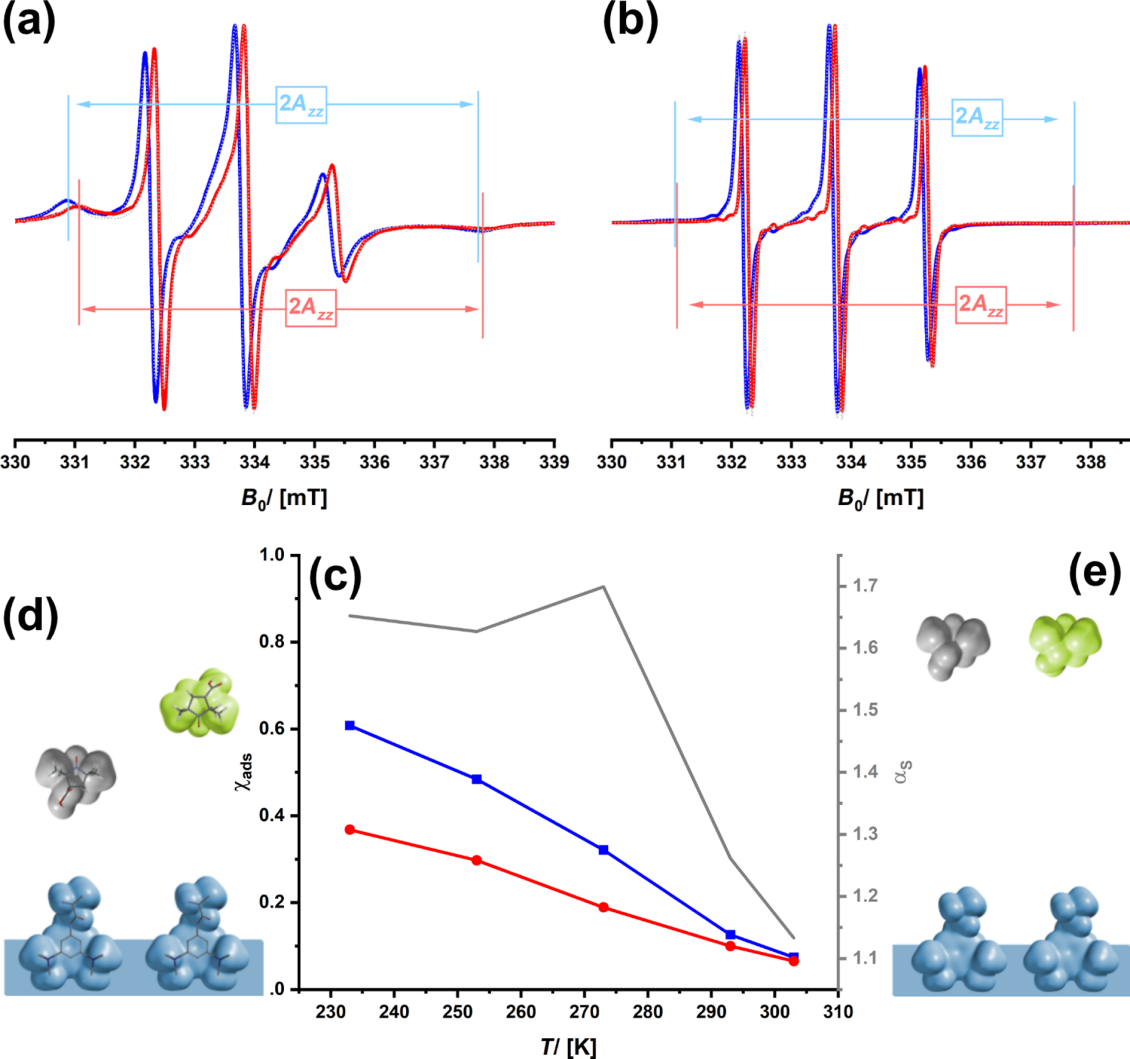
cw-ESR spectra of (+)-3CP (blue) and (−)-3CP (red) dissolved in ethanol and confined in SerNH_10_oSIL recorded at *T* = 213 K (a) and *T* = 293 K (b). Grey, dotted lines show the result of the fit of the spectra. (c) Temperature-dependence of the fraction of adsorbed species (left axis, black) and the selectivity factor (right axis, grey). Visualization of the situation at the interface. (d) A difference in adsorption (one enantiomer shows a higher fraction of the immobile species such as in spectrum (a); Δχ_ads_ is large) and a difference in the 2*A_zz_* value, which contains information about the local environment. This is, in turn, connected to high(er) selectivity values α_s_. (e) In contrast, a case in which both species are equal and mobile (such as in spectrum (b)) and exhibit no selectivity.

Figure 4 shows the cw-ESR spectra of the two spin probes infiltrated into SerNH_10_oSil at two different temperatures; the entire temperature series is shown in Supporting Information File 1, Figure S11. At *T* = 213 K, there is a substantial amount of the immobile spin probe (Figure 4a), which decreases with higher temperature until almost all spin probes are mobile at room temperature (Figure 4b). The interaction enthalpy of the spin probe with the pore walls of the aerogel is relatively low, and, together with the relatively large size of the pores, a higher temperature easily induces detachment from the surfaces. If one compares (+)-3CP and (−)-3CP, one sees differences. More of (+)-3CP is attached to the surfaces at *T* = 213 K, which can be explained by the diastereoselective interaction with the chiral amino acid groups. One can calculate a selectivity α_s_, defined as the ratio of the spin probes in their immobile state, χ_ads_. α_s_ has dropped to almost 1.0 at *T* = 293 K, which can be explained because neither of the enantiomers interacts with the pores, but is now fully dissolved in the solvent.

**Figure 5:**
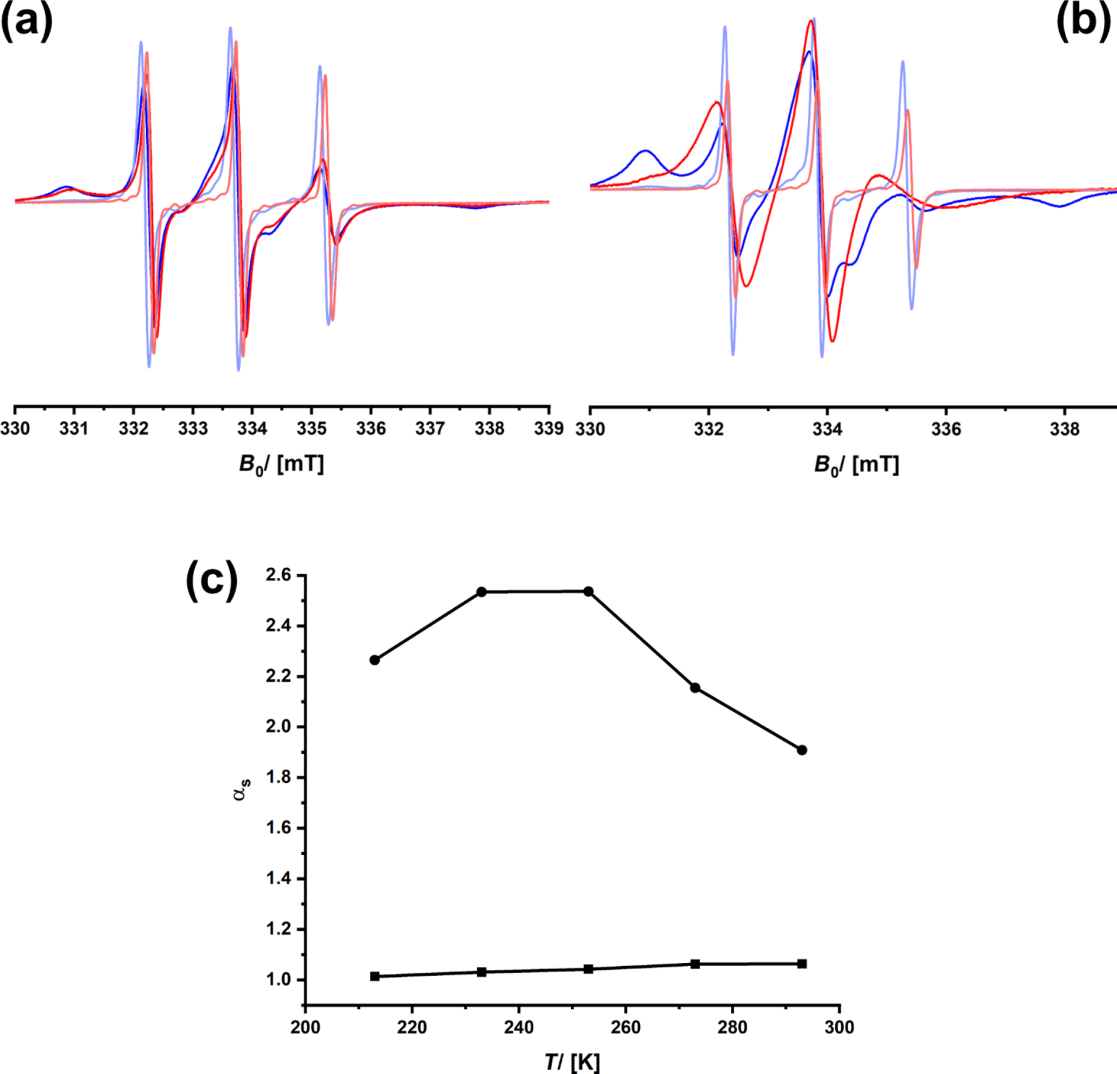
cw-ESR spectra of (+)-3CP (blue) and (−)-3CP (red) confined in SerNH_10_SIL recorded at *T* = 213 K (dark colors) and *T* = 293 K (pale colors); the solvents are ethanol (a) or isopropanol (b). (c) Temperature dependency of the selectivity for ethanol (squares) or isopropanol (circles) as solvents.

Next, we checked if SerNH_10_Sil (Figure 1) shows the same behavior. The data are shown in Figure 5. It can be seen that the ESR spectra of both enantiomers look almost the same at all temperatures. Despite there being a significant amount of surface-adsorbed species (≈70%), there is no differentiation between (+)- and (−)-3CP, meaning that also α_s_ ≈ 1. The latter result is astonishing because both SerNH_10_Sil and SerNH_10_oSil contain the same amino acid and the same overall concentration of it.

**Figure 6:**
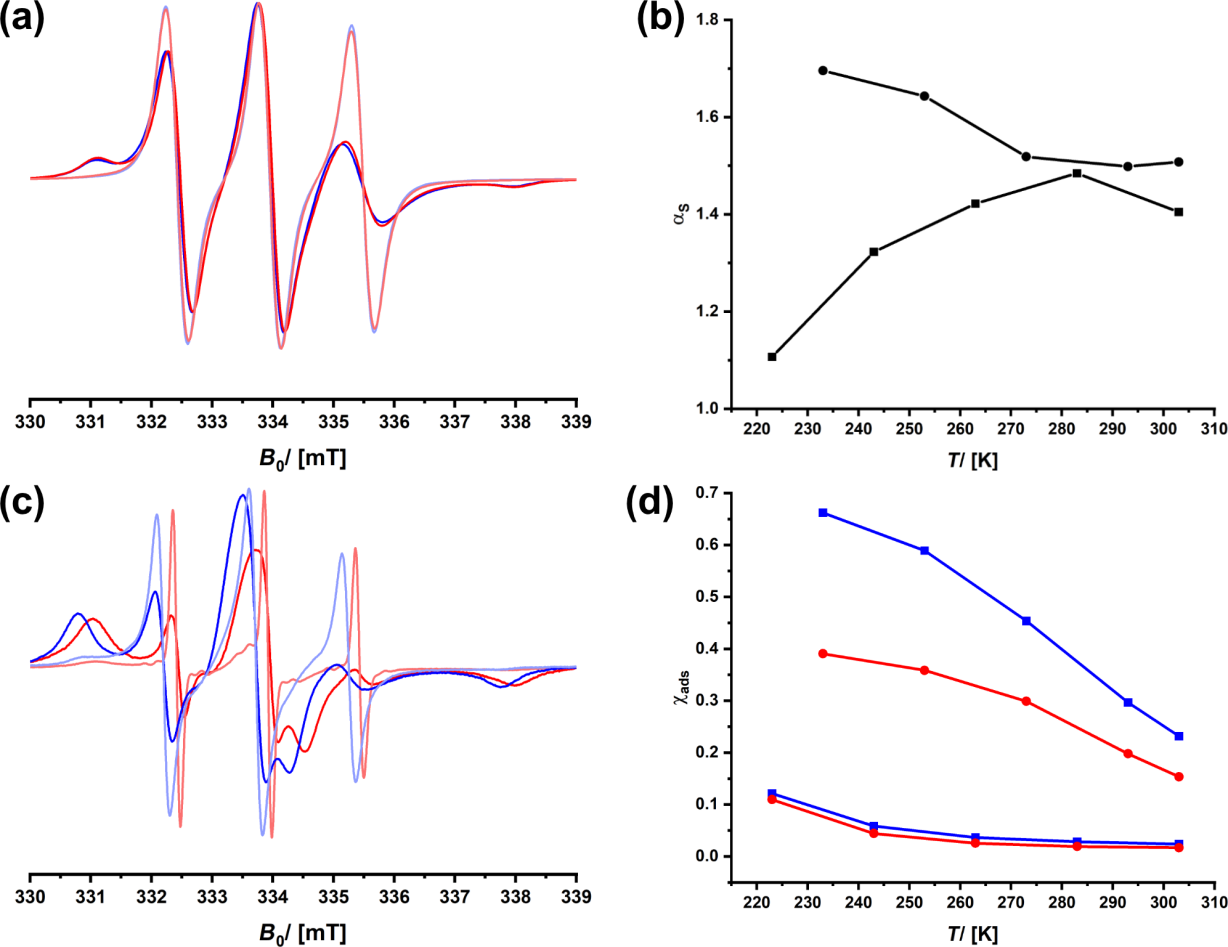
(a) cw-ESR spectra of (+)-3CP (blue) and (−)-3CP (red) confined in AlaNH_10_oSIL recorded at *T* = 223 K (dark colors) and *T* = 293 K (pale colors); the solvent is ethanol. (b) Temperature dependency of the selectivity for ethanol (squares) or isopropanol (circles) as solvents. (c) cw-ESR spectra of (+)-3CP (blue) and (−)-3CP (red) confined in AlaNH_10_oSIL recorded at *T* = 233 K (solid colors) and *T* = 293 K (pale colors); the solvent is isopropanol. (d) Temperature dependency of the ratio of the adsorbed species.

Therefore, we investigated another material that contains an alternative amino acid, that is, alanine (AlaNH_10_oSil). The cw-ESR spectra recorded in ethanol as solvent are shown in Figure 6a. One can see that, at high and at low temperatures, there are barely any differences between the spectra of (+)-3CP and (−)-3CP. If there is any stereoselectivity, it is highly reduced. Quantitative evaluation of the ESR spectra, determination of the ratio of the adsorbed species, and calculation of α_s_ confirm this (Figure 6b). Surprisingly, the selectivity seems to raise with increasing temperature and reaches a maximum at *T* = 293 K, although the immobile/surface-attached component is missing almost completely in the ESR spectra. One can conclude that it is problematic to consider only α_s_ and that χ_ads_ needs to be considered at the same time (Figure 6d). Consistent with the ESR spectra, one sees that the fraction of the spin probes’ interaction with the surface of the aerogel is very low even at low temperatures and then decays further. Because the low amount of spin probe molecules interacting with the surfaces is not considered in α_s_, it can be misleading if one only looks at the selectivity parameter. There is only one explanation why χ_ads_ is so low in AlaNH_10_oSil. It is much more preferable for both enantiomers of 3CP to interact with the solvent (ethanol) than with the surfaces. If there is no dynamic equilibrium between a substantial fraction of the spin probes interacting with the surfaces, there cannot be a sufficient differentiation between (+)-3CP and (−)-3CP, even if chiral functionalities are present at the pore walls. There are two limiting cases: The guest molecule confined to a porous host only interacts with the solvent. Then, its diffusion rate is no longer influenced by the nature of the pore surfaces. Alternatively, if the guest–surface interaction is predominant, the spin probe is immobilized (i.e., chemisorption) and does not move at all. Neither scenario is suitable for differentiating between two chemically similar spin probe molecules moving through the pore system. The ideal situation is between these two limiting cases; there is a significant but not too high fraction of the guest molecules interacting with the pore walls. In the case of SerNH_10_oSil (Figure 4), we could observe χ_ads_ values up to 0.6, meaning that the α_s_ values are also more reliable. Obviously, SerNH_10_oSil interacts stronger with 3CP than AlaNH_10_oSil, but also not too much.

Of course, one could now adjust numerous materials parameters for altering χ_ads_ and then look at how this affects α_s_. However, it is much simpler to change the solvent first as this, according to [42,44], alters the difference in the interaction profiles. Isopropanol (ε = 18.3, μ = 1.66 D) has a lower dielectric constant ε and lower dipole moment μ than ethanol (ε = 24.2, μ = 1.69 D). Because of the carboxyl group in 3CP (see Figure 3), together with the dipole moment of the N–O bond, one can understand the tendency for good interaction with polar solvents. Thus, we assume that the higher hydrophobicity of isopropanol leads to enhanced interactions with the pore surfaces. The ESR spectra of 3CP dissolved in isopropanol and confined to SerNH_10_SIL (Figure 5b) and AlaNH_10_oSIL (Figure 6c) are shown. One sees a marked effect: As expected, the amount of the immobile spin probe has become much higher and is up to 0.7 in the case of (+)-3CP in AlaNH_10_oSIL (Figure 6d). The selectivity values are higher now, and, in comparison, the effect is even more pronounced for SerNH_10_SIL where values higher than α_s_ = 2 are reached.

### Investigations on an independent chiral matrix

**Figure 7:**
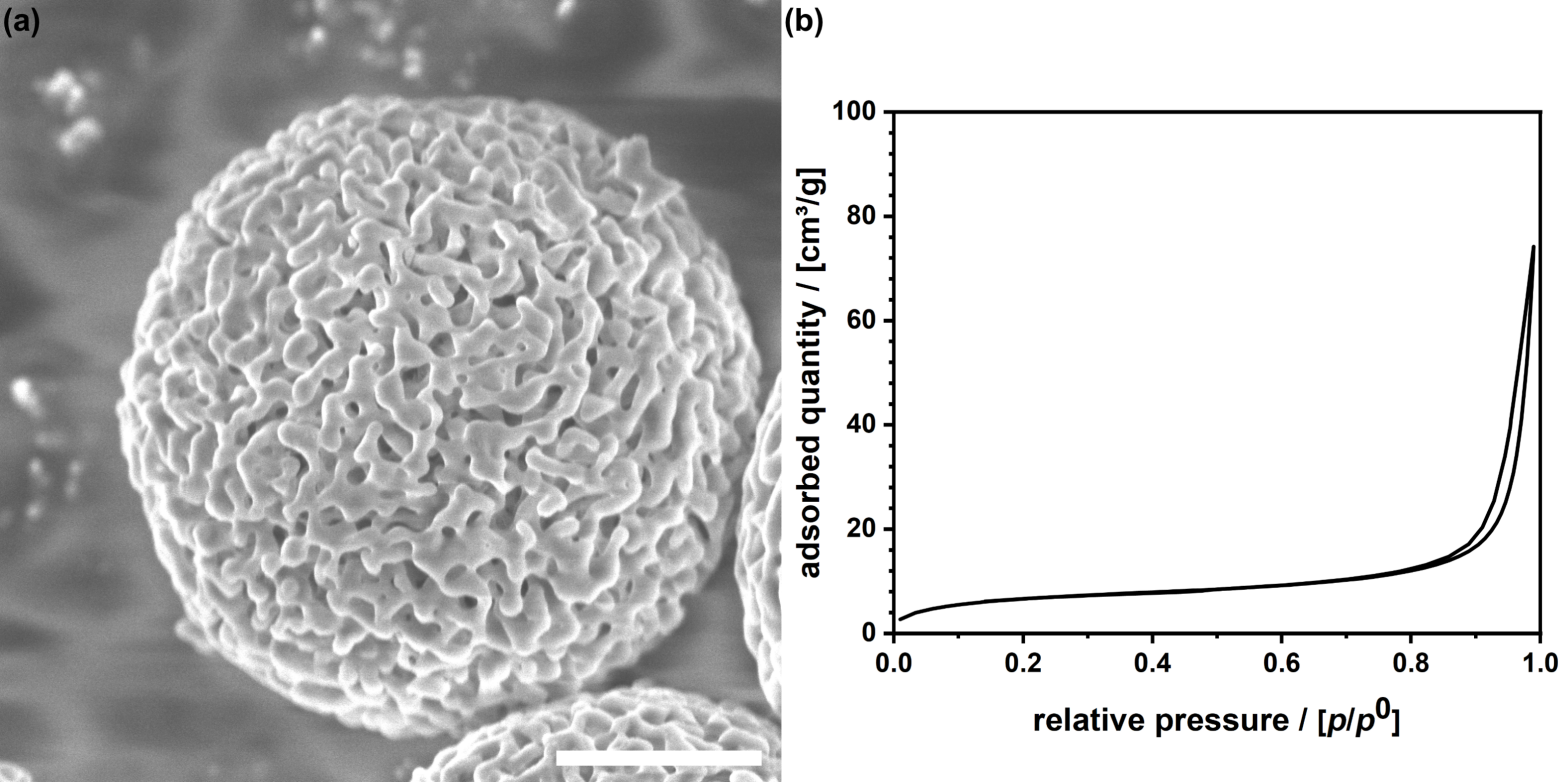
(a) SEM micrograph of a single porous particle of the commercial Chiralpak IG-3 material; scale bar = 500 nm. (b) N_2_ physisorption isotherm.

Chiralpak IG-3 is a commercially available type of chiral stationary phase used in HPLC for the separation of enantiomers [66]. Chiralpak IG-3 consists of a chiral selector that is based on cellulose derivatives (amylose). These derivatives are typically chemically bonded to a silica gel support. Chiralpak IG-3 consists of spherical silica particles with a macroporous internal structure that resembles the structure of aerogels quite well (Figure 7a). Other than aerogel monoliths, the packing of the polydisperse particles leads to a significant portion of large interparticle voids. However, porosity and specific surface area (*A*_BET_ = 25 m^2^·g^−1^) are lower as determined from N_2_ physisorption analysis (Figure 7b).

**Figure 8:**
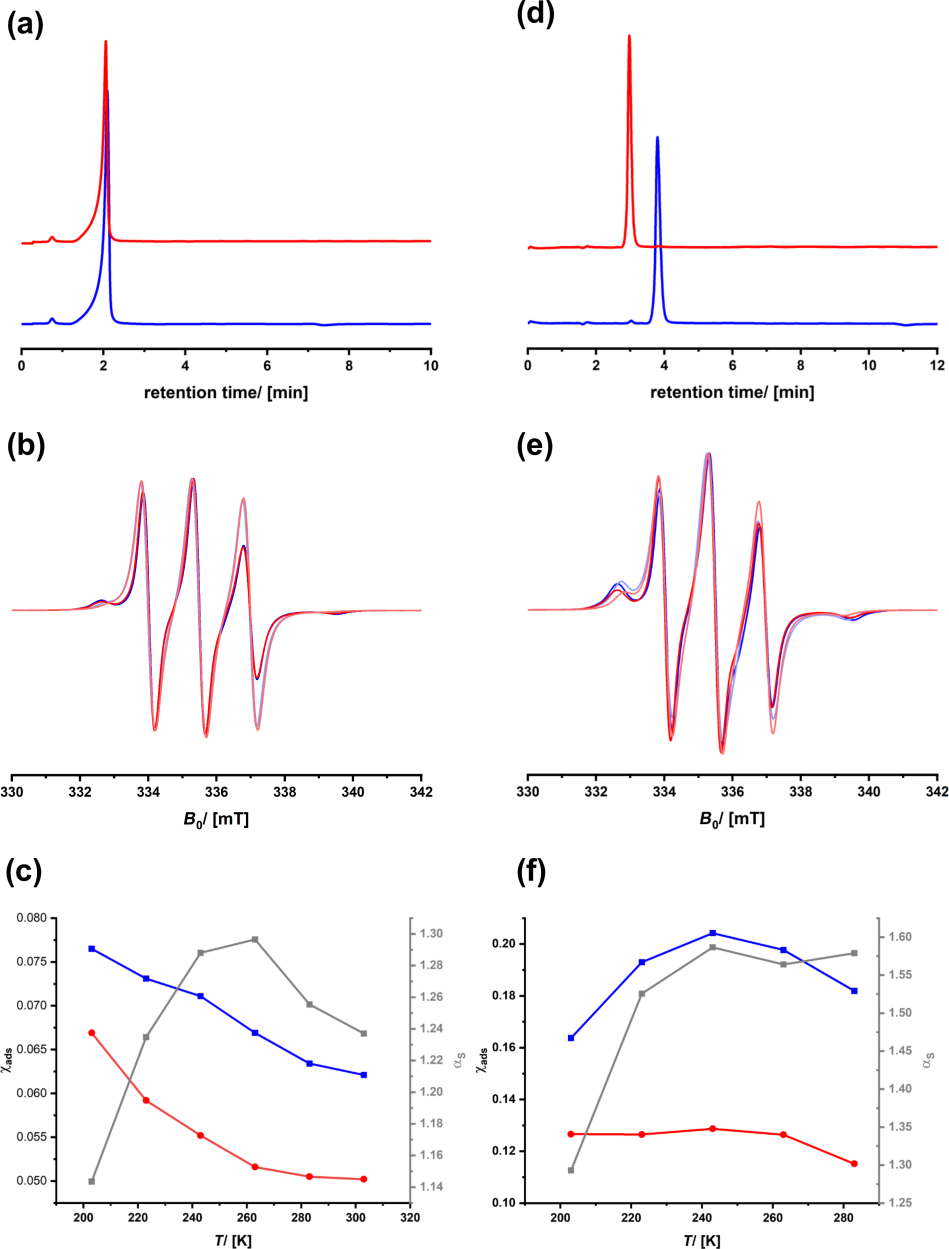
HPLC chromatograms using Chiralpak IG-3 as a stationary phase of (+)-3CP (blue) and (−)-3CP (red) using ethanol (a) or an 80:20 mixture of pentane and ethanol (d) as solvent. cw-ESR spectra of (+)-3CP (blue) and (−)-3CP (red) confined in Chiralpak IG-3 recorded at *T* = 203 K (dark colors) and *T* = 293 K (pale colors); the solvent is either ethanol (b) or an 80:20 mixture of pentane and ethanol (e). Temperature dependency of the selectivity (grey graphs) and the fraction of the adsorbed species of (+)-3CP (blue) and (−)-3CP (red); the solvent is either ethanol (c) or an 80:20 mixture of pentane and ethanol (f).

We applied the material in HPLC and performed two runs, that is, one with (+)-3CP and one with (−)-3CP. The chromatograms recorded in ethanol are shown in Figure 8a. Both enantiomers elute at the same retention time, meaning that separation is impossible under these conditions. The cw-ESR spectra recorded using ethanol as a solvent are shown in Figure 8b. There are minor differences in the spectra of (+)- and (−)-3CP, and, as a result, the selectivity factor remains small (Figure 8c). One also sees that the fraction of the species interacting with the surfaces is small (<10%). Because ethanol predominantly interacts with 3CP, the contact of the spin probe with the chiral groups on the surface is insufficient.

A less polar solvent system, comprised of a mixture of ethanol and pentane (20:80), was used next, and all measurements were repeated. One sees a marked difference in the ESR spectra and the parameters derived from Figure 8e,f. The contribution of the immobile species has increased, so has the difference in interaction with the surfaces, which leads to a selectivity factor of above 1.5. Most importantly, we now see in HPLC a quantitative separation of (+)- and (−)-3CP. One can learn from the mentioned results that ESR spectroscopy can also be applied to commercial materials to learn about the factors determining efficiency in chiral chromatography. Obviously, a selectivity factor of more than 1.5 combined with a fraction of molecular species interacting with the surfaces of the stationary phase of the order of 20% is sufficient to reach satisfactory separation results.

Interestingly, one sees a repeated feature for the conditions under which enantioselection is possible. The selectivity curves go through a maximum which is at ≈270 K for SerNH_10_oSIL (Figure 4c), at ≈250 K for SerNH_10_SIL (Figure 5c), at ≈280 K for AlaNH_10_oSIL (Figure 6b), and at ≈240 K for Chiralpak IG-3 (Figure 8f). The thermal energy at *T* = 260 K is approximately 2 kJ·mol^−1^. The difference in interaction enthalpies of two enantiomers with a chiral surface depends on many factors, but it is exactly in the range of 1–2 kJ·mol^−1^ [18,67], which we could deduce from ESR spectroscopy.

If (+)-3CP is more frequently interacting with the chiral surface than (−)-3CP, the fraction of the immobile component is higher and its transport through the material is slower, which leads to separation and a potentially promising material for chiral chromatography. The location of a spin probe is impossible to determine from cw-ESR, and special spin-echo measurements are necessary, as we reported in [46]. However, the polarity of the environment of a nitroxide spin probe such as 3CP influences the spatial distribution of the electron in the N–O bond and, therefore, the hyperfine coupling with the ^15^N nucleus [68–69]. The spectroscopic signature is the *A_zz_* parameter (see Figure 4). Because a polar environment favors the charge-separated mesomeric structure, where the electron is located at the nitrogen atom, *A_zz_* increases. If the two enantiomers (+)- and (−)-3CP, in average, experience a different environment, for instance, because one is more frequently bound to the surface of the chiral host, one should expect a difference, Δ*A_zz_*. Therefore, we re-evaluated the data of all materials and checked whether there is a correlation of Δ*A_zz_* with the selectivity.

**Figure 9:**
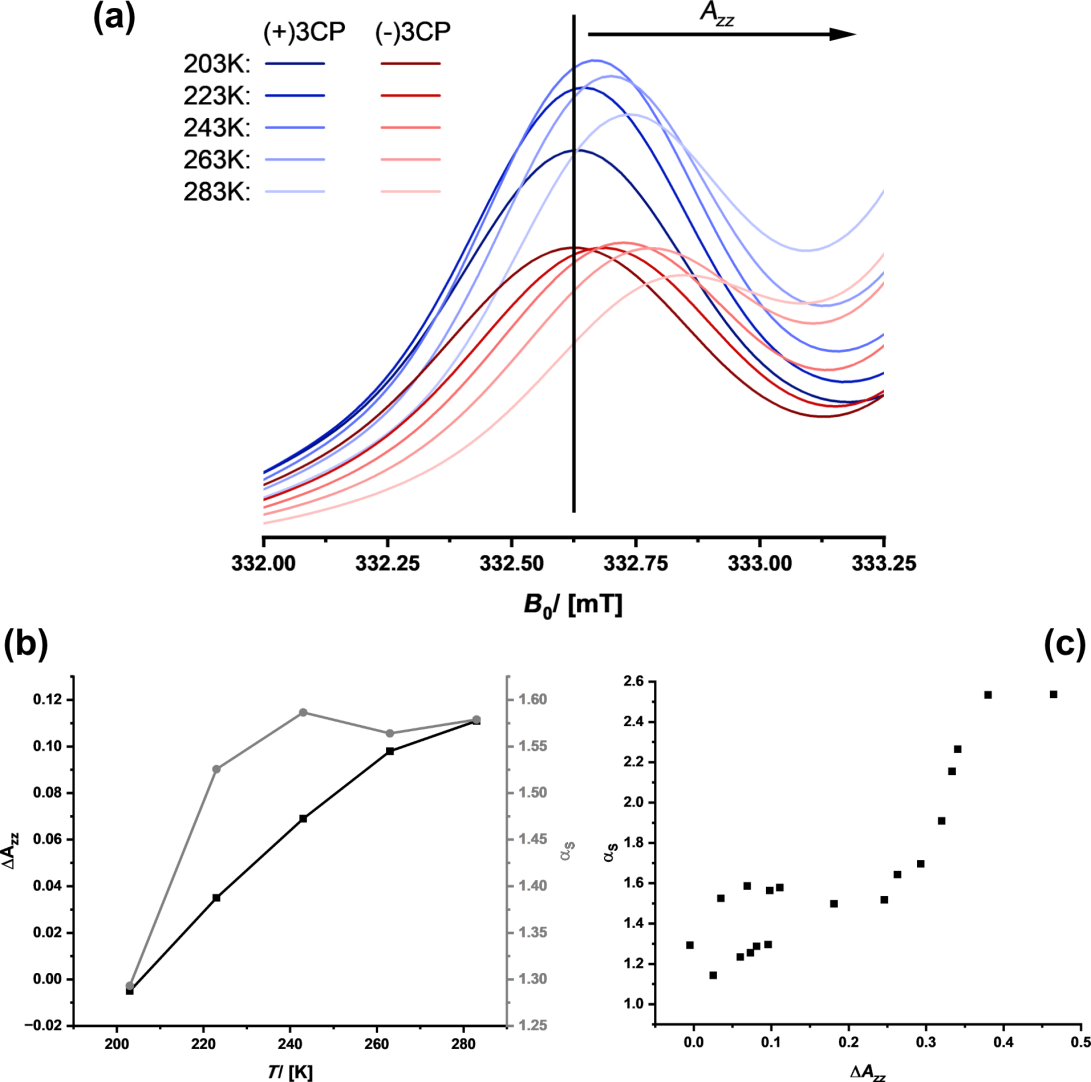
(a) Enlargement of the region of the ESR spectra of the spin probes dissolved in the ethanol/ pentane mixture confined in Chiralpak IG-3. (b) Temperature dependency of the selectivity factor (grey circles) compared to the difference in the *A_zz_* shift of the slow constituent. (c) Potential correlation between Δ*A_zz_* and α_s_ for all samples for which *A_zz_* could be determined for the slow component.

Figure 9a shows the region of the low field signal of (+)- and (−)-3CP dissolved in ethanol/pentane and inside Chiralpak IG-3, that is, for the situation for which we know that there is successful stereoselection even at room temperature. One sees how the position of the signal shifts to higher fields with increasing temperature because the spin probes can detach from the surface, which will change the local environment. This shift contributes to the *A_zz_* value. One also sees that there are differences between (+)-3CP and (−)-3CP, which is reasonable, assuming that their interaction with the chiral surface is also different. Therefore, the difference in *A_zz_* values, Δ*A_zz_*, is indicative of subtle variations in the chemical surroundings of both spin probes. The Δ*A_zz_* values are indeed small, which would confirm the assumption that the difference in interaction potentials is only of the order of 1–2 kJ·mol^−1^. However, if one plots the temperature dependency of Δ*A_zz_* (Figure 9b), it qualitatively matches the temperature dependency of the α_s_ value.

Unfortunately, it was not possible to determine *A_zz_* values for all samples because the intensity of the pattern at high field was too low in intensity, for example, at higher temperatures (see Figure 4b). However, for the remaining samples, Figure 9c indicates that there might indeed be a correlation between *A_zz_* and α_s_. This would mean that the higher the enantioselectivity is, the more different are the chemical environments that the two spin probes, (+)- and (−)-3CP, experience.

While we can learn a lot about the behavior of chiral guests inside chiral porous host materials and correlate the ESR results to chromatography phenomena, it is important to emphasize that the spectra were recorded under static conditions. In contrast, HPLC operates under dynamic flow using analyte mixtures.

### Neighboring group effects

The choice of solvent is obviously a way to activate the difference in the interaction potentials. The latter reflects how one performs chromatography, which typically uses a gradient of solvents. Often, one starts with a hydrophobic solvent, and then the polarity of the mobile phase becomes more and more polar. Eventually, one reaches a spot where one component moves faster through the column than the other. However, chromatography columns are highly engineered materials that demonstrate the importance of the chemical and structural architecture of the stationary phase. Therefore, it is an important question to consider which possibilities exist to tune the interaction of guest molecules with the surfaces more precisely.

Because the concentration of the amino acid groups is relatively low in our materials, the first idea is to increase it. The measurement results using AlaNH_15_oSIL are shown in Supporting Information File 1, Figure S12. The selectivity factor is in the same range as for AlaNH_10_oSil. Thus, a higher degree of amino-acid modification does not bring substantial improvement, which we did not expect.

**Figure 10:**
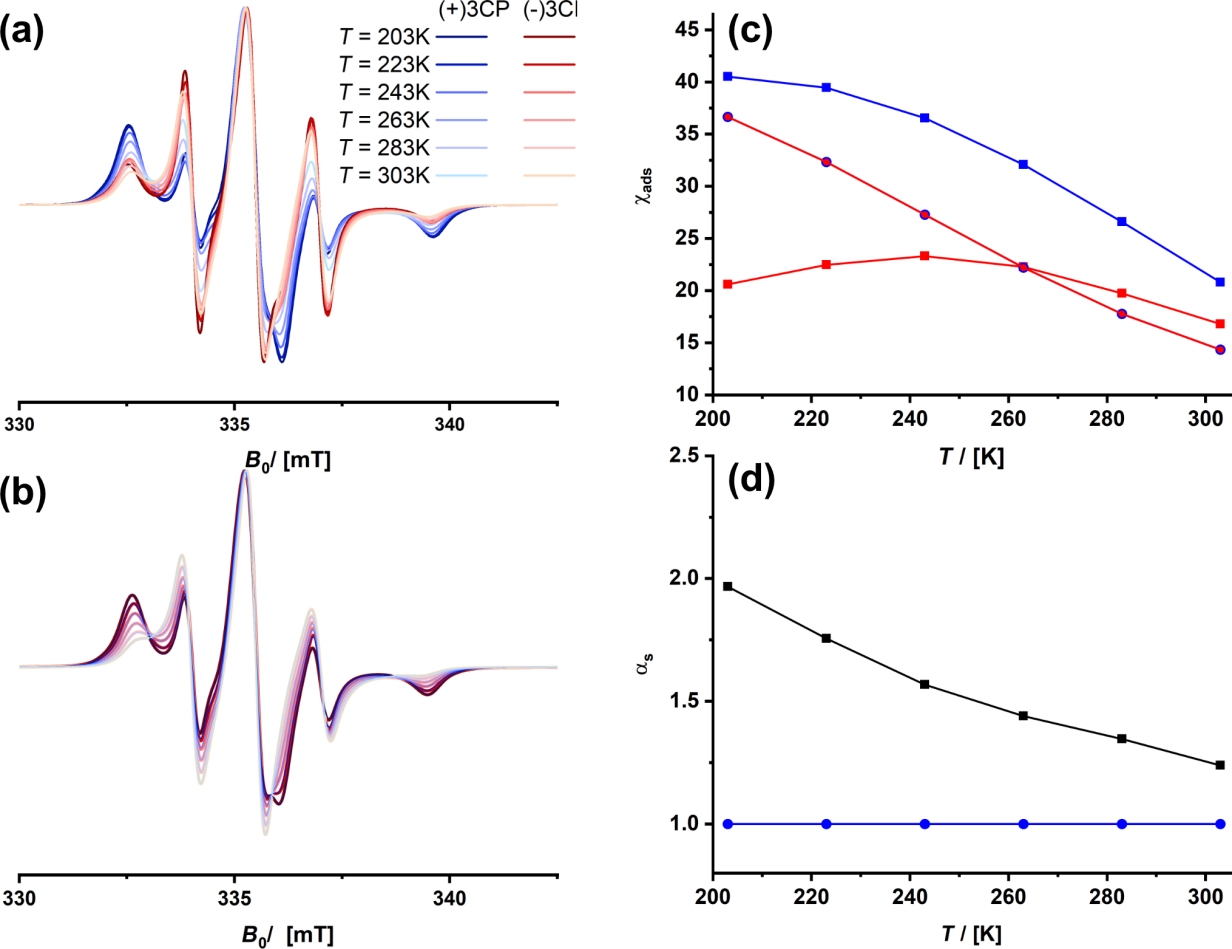
cw-ESR spectra recorded at different temperatures for SH-FmocAlaNH_10_oSIL (a) and SH-AlaNH_10_oSIL (b). Temperature dependency of χ_ads_ (c) and selectivity factor (d) of (+)-3CP (blue) and (−)-3CP (red) in FmocAlaNH_10_oSIL (squares) and SH-AlaNH_10_oSIL (circles).

Therefore, we concentrate now on the samples that were prepared to introduce neighboring groups (NGs, see Scheme 1 and Scheme 2). Figure 10 shows the data for the alanine-containing organosilica materials before and after detachment of the protecting group. The effects are astonishing. Although the chiral selector is present in both materials, there is no stereoselective discrimination for SH-AlaNH_10_oSIL at all. Although there is a significant amount of adsorbed spin probes at all temperatures, the ESR spectra of (+)-3CP and (−)-3CP are almost identical. Thus, the selectivity factor is 1, meaning there is no selectivity. These results again illustrate that the spin probes must experience a slightly different environment. One of the best selectivity factors, which even outmatches the commercial chromatography material Chiralpak IG-3, however, was found for FmocAlaNH_10_oSIL. The bulky Fmoc protecting group, which makes the surfaces of the material much more hydrophobic, significantly modulates the interaction with the spin probes and boosts selectivity.

**Figure 11:**
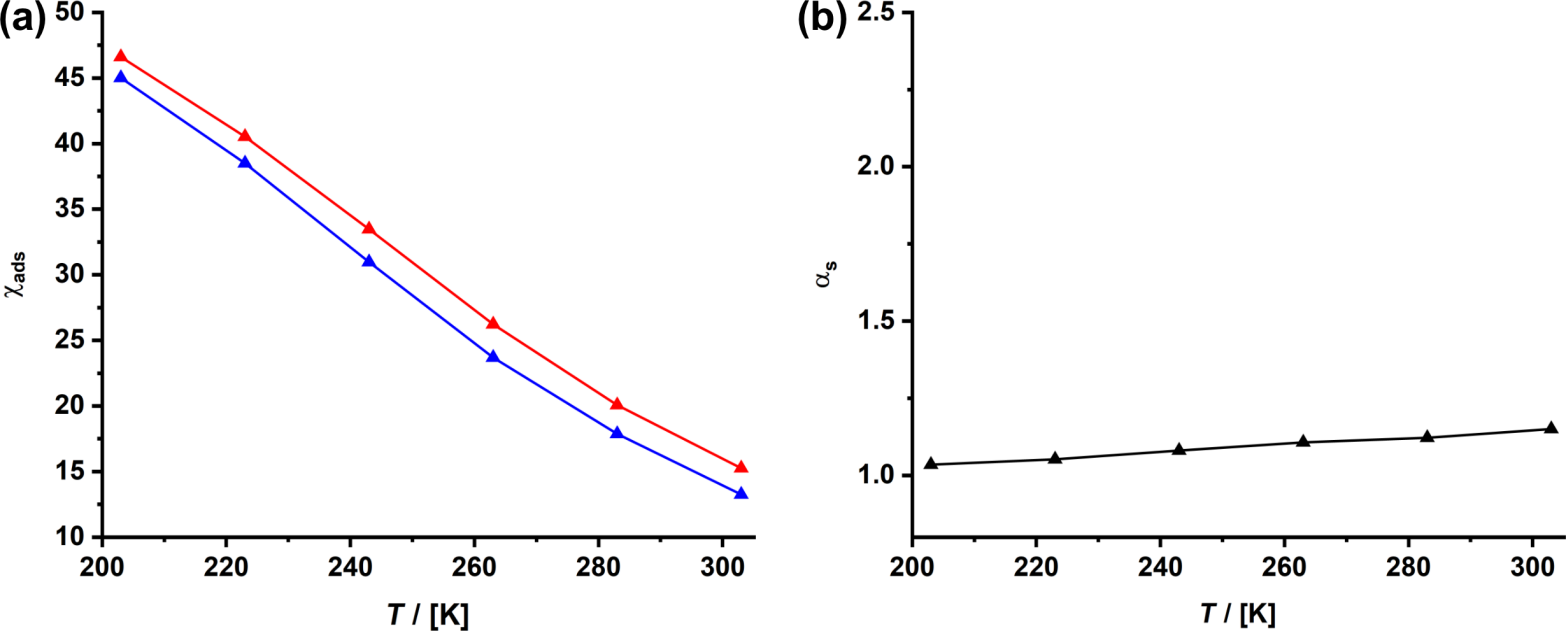
Temperature dependency of χ_ads_ (a) and selectivity factor (b) of (+)-3CP (blue) and (−)-3CP (red) in Ar_F_SH-AlaNH_10_oSIL.

Investigating the ArFSH-AlaNH_10_oSil material probed whether only a more hydrophobic surface leads to pronounced selectivity. The perfluorophenyl ring is also very bulky. Yet, the results were disappointing. Although there is again significant adsorption of the spin probes at all temperatures, there is only a minor difference for the two enantiomers (Figure 11, ESR spectra are shown in Supporting Information File 1, Figure S12).

**Figure 12:**
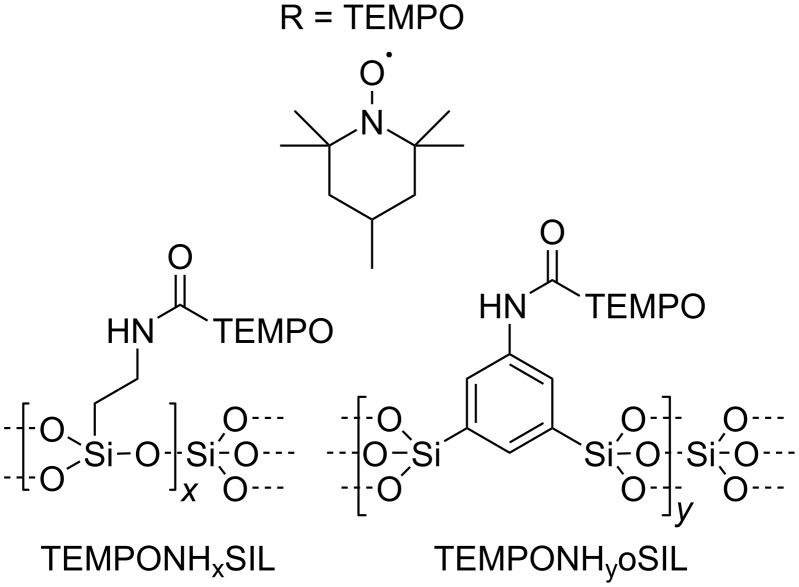
Organosilica materials containing a spin probe attached to the surfaces.

For understanding the different and unexpected behavior of the SIL compared to the oSIL materials (Scheme 2), we prepared two new materials, namely, TEMPONH*_x_*SIL and TEMPONH*_y_*oSIL (Figure 12). Via amide coupling chemistry, in a post-functionalization step, we attached a pentafluorophenol-activated 4-carboxy-2,2,6,6-tetramethylpiperidinyloxyl (R = TEMPO; Figure 12) group. The reaction delivers an ESR-active spin probe covalently linked to the surface of the aerogels.

**Figure 13:**
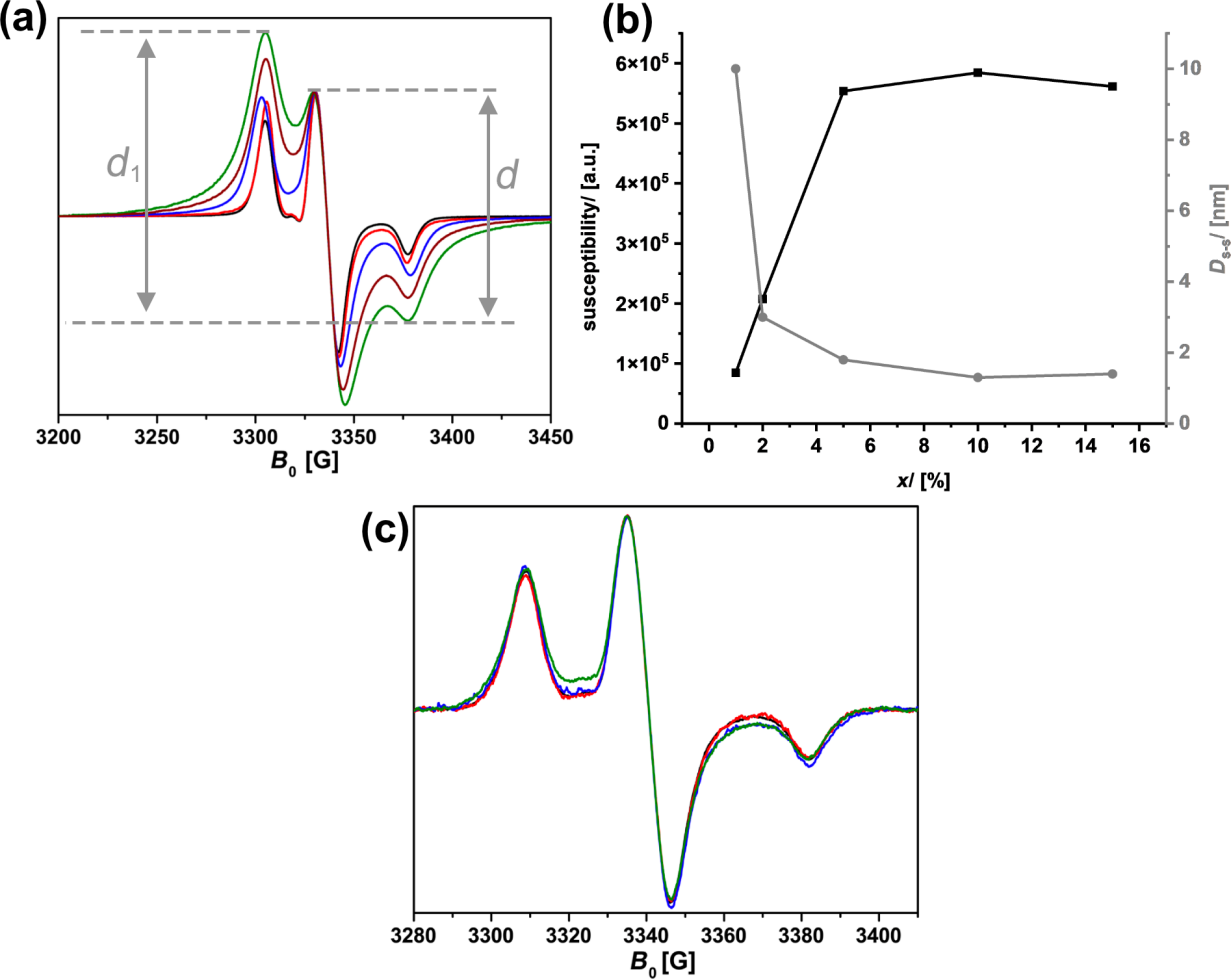
(a) ESR spectra recorded at *T* = 103 K for TEMPONH*_x_*SIL with *x* = 0.01 (black), 0.02 (red), 0.05 (blue), 0.1 (green), and 0.15 (brown). (b) Magnetic susceptibility and the distance between the spin probes *D*_s–s_ for the different samples. (c) ESR spectra recorded at *T* = 103 K for TEMPONH*_y_*oSIL with *x* = 0.01 (black), 0.02 (red), 0.05 (blue), and 0.1 (green).

Figure 13a shows ESR spectra of TEMPONH*_x_*SIL with *x* increasing from 0.01 to 0.15, recorded at 103 K. Because of the attachment to the surface and due to the low temperature, the spectra are characteristic for a slow tumbling dynamics and the solid-state situation of the spin probe. In ESR, one always plots the first derivative of absorption spectra, which means that the magnetic susceptibility χ is proportional to the second integral of the curves, that is, proportional to the area of absorption signals. No correlation exists between χ and the available amine groups *x* in TEMPONH*_x_*SIL (Figure 13b). The curve saturates at *x* > 0.05, and there are two explanations for this result. First, because of the co-condensation route, not all amine groups are located at the surface, but some will be buried in the volume of the pores. Second, once the surface of the pore walls is saturated with R, no further modification is possible. The latter will presumably also correlate to the steric hindrance of R. A relatively bulky molecule, such as TEMPO, seems to block a lot of space on the surface. It is well known that the ESR signals of two paramagnetic centers are influenced by dipolar coupling if these paramagnetic centers are close to each other (<10 nm). In the case of nitroxide spin probes, the theory developed by Ionita et al. [70] allows one to estimate the distance *D*_s–s_ between the two spins from the ratio *d*_1_/*d* in the ESR spectra (Figure 13a). One sees in Figure 13b that already for *x* > 0.05, the distance between the TEMPO units is around 1.4 nm. One may assume that a larger number of amines/*x* does not necessarily lead to a higher degree of functionalization in the post-functionalization step.

The introduction of phenyl spacers in the oSIL materials is expected to lead to a larger distance between the amine functionalities. The latter assumption is confirmed by the ESR measurements performed on TEMPONH*_y_*oSIL. The spectrum for a material containing 10% of the amine groups looks the same as for the lower functionalization degrees and indicates a distance of ≈4 nm between the surface-attached radicals. It needs to be mentioned that the method of Ionita et al. [70] is sensitive only for very small distances below 5 nm; if distances between the spins are larger, the degree of dipolar coupling is too small to lead to notable changes in the ESR spectrum. This situation is exactly what we see in Figure 13c.

The latter measurements reveal that the distance between the chiral center (amino acid) and a neighboring group attached to the organosilica network of R_NG_SH-R*NH*_z_*OSIL aerogel samples is presumably too large to have an effect, which makes it difficult to exploit neighboring group effects for this particular type of material. Here, the simpler R*NH*_x_*SIL material seems to have advantages because the functional groups are much closer.

## Conclusion

The paper demonstrates that observing the rotational dynamics of organic nitroxides as spin probes in ESR spectroscopy is a powerful technique for investigating the interactions and interplay of factors relevant for transport in chiral chromatography and the interactions of the surfaces of a solid phase with the enantiomers.

A number of important results could be obtained using a commercially available benchmark material. Not only is it possible to use the ESR method on powdered samples, but one could also see that the proper choice of a solvent is crucial for reaching enantiomer separation. Despite chiral surfaces, if the guest species predominantly interacts with the solvent, there is no separation, which is reflected by the similarity in the dynamics of the spin probe. With a set of custom-made organosilica materials, it was possible to specify what can be understood as a proper interaction. As expected, a dynamic equilibrium between surface-adsorbed and mobile guest species is necessary. This also means that the interaction with the surfaces of the porous host must not be too strong because both enantiomers would be immobilized then. Temperature-dependent measurements have reflected that the stereodifferentiation in the interaction should be around 1.5 kJ·mol^−1^. We report cases in which there is a seemingly perfect degree of adsorption, not too high, not too low. Still, the selectivity values remain close to 1, meaning that there is no separation. An advantage of the ESR spectroscopy is that the spectra contain information about the local environment of the spin probe. We found a correlation between the enantioselectivity and the difference in environment between the two enantiomers. This means that notable selectivities larger than 1.5 can only be realized if there are subtle differences in the environment of the two spin probes.

Materials were presented that display much higher selectivity values than the commercial material. In particular, first promising results show that a neighboring group attached near the stereocenter can boost the selectivities.

## Experimental

### Syntheses

Unless stated otherwise, syntheses were performed using general Schlenk techniques under a nitrogen atmosphere. The solvents were dried according to the standard literature and stored under argon. All starting materials used for the synthesis were purchased from commercial sources (Merck or TCI).

**SIL:** In a snap lid vial, dissolve 2.0 mL tetramethoxysilane (2.04 g, 13.4 mmol, 1 equiv) in 1.5 mL methanol (p.a.). With vigorous stirring, a solution of 1.0 mL NH_3_ (55.6 mmol H_2_O, 4 equiv) in 1.5 mL methanol (p.a.) is quickly added and stirred for 1 min. As soon as the gel is completely formed, it is left to age at room temperature. After ageing, the gel is layered with acetone (p.a.), and the acetone is changed five times after several hours. This is followed by supercritical drying with CO_2_.

**NH_10_SIL:** Dissolve 0.11 g urea (1.83 mmol) with 0.095 g PEG (*M*_w_ = 10000 Da) in 0.875 mL 0.56 M acetic acid. Then 0.375 mL of an acetic acid/acetate buffer is added, and the solution is cooled to 4 °C for 20 min. Add 0.64 g tetramethyl orthosilicate (4.23 mmol) and 0.082 g (3-aminopropyl)triethoxysilane (0.46 mmol) and stir at 4 °C for further 20 min. The solution is now transferred (if desired) to a container of any shape and allowed to harden at room temperature. It is overcoated with triethylamine for 2 d, and then the solvent is statically exchanged with acetone. An aerogel monolith is obtained with a size corresponding to the volume of reagents used. The material can be dried supercritically in CO_2_ for obtaining an aerogel.

^29^Si NMR (79.5 MHz, spin echo [*d*_1_ = 350s], 10 kHz) δ −58.4 (T1), −68.3 (T2), −80.0 (T3), −92.4 (Q2), −100.2 (Q3), −109.1 (Q4); ^13^C NMR (100.6 MHz, cp [*d*_1_ = 10], 10 kHz) δ 10.4 (C1), 35.7 (C2), 52.4 (C3) of the Si–C1H_2_–C2H_2_–C3H_2_–NH_2_ chain.

**3,5-Bis(triisopropoxysilyl)thiophenol:** The sol–gel precursor was prepared and characterized as described in reference [63].

**SHoSil:** In a typical synthesis, 0.22 mmol of 1,3-bis(triisopropoxysilyl)thiophenol is dissolved in 2 mL of ethanol. 50 µL of a 1 M HCl solution is added under vigorous stirring. After hydrolysis for 1 h, 50 µL of concentrated ammonia was added, and the solution was filled into a syringe and gelled overnight. Aerogels can be obtained after solvent exchange followed by supercritical drying with CO_2_.

**Fmoc-Ser-OPfp:** 5.0 g of Fmoc-ʟ-serine-OH (15.28 mmol) was diluted in ethyl acetate and cooled down to 0 °C. 2.81 g pentafluorophenol (15.28 mmol) was added and stirred for 30 min. Now, 3.15 g dicyclohexyldicarbodiimide (15.28 mmol) was added. After 15 min, the solution was allowed to warm up to room temperature, and stirring was continued overnight. The precipitate was separated, and the solvent was removed in vacuum yielding 6.4 g (12.97 mmol; 85%) of the desired product.

^1^H NMR (400.1 MHz, CDCl_3_) δ 2.61 (s, 1H, Ser-OH), 4.03 and 4.22 (dd, 1H each, Ser-CH_2_), 4.24 (t, 1H, Fmoc-CH), 4.46 (m, 2H, Fmoc-CH2), 4.82 (m, 1H, Ser-CH); 5.93 (d, 1H, NH); 7.30 (dt separated by 1.26 Hz, ^3^*J* = 7.48 Hz, 2H), 7.39 (dt separated by 0.54 Hz, 2H, ^3^*J* = 7.52 Hz), 7.52 (m, 2H), 7.76 (d, ^3^*J* = 7.46 Hz, 1H), 7.51(d, 2H, ^3^*J* = 7.55 Hz) (Fmoc–H_arom_); ^13^C NMR (100.6 MHz, CDCl_3_) δ 47.2 (Fmoc–CH); 56.0 (Ser–CH); 63.0 (Ser–CH2); 67.6 (Fmoc–CH2); 120.1, 125.1, 127.2, 127.9 (Fmoc–CH_arom_); 141.41, 141.45 (Fmoc–*tert*-C); 143.6, 143.7 (Fmoc–*tert*-C); 156.3 (Fmoc–CO); 167.1 (Ser–CO).

**Fmoc-Ala-OPfp:** The synthesis was performed analogous to Fmoc-Ser-OPfp.

**SerNH_10_SIL:** 0.25 g 10% NH_10_SIL is overlaid with a solution of 0.39 g Fmoc-Ser-OPfp in 2 mL THF (*n* = 0.79) and 0.5 mL of a 10 wt % *N*,*N*-diisopropylethylamine in THF is added. It is reacted for 2 d at room temperature. The solution is replaced by THF and then a solution of 20% (*v*/*v*) piperidine in THF is infiltrated. After 2 d, the supernatant solution is removed and replaced several times with acetone and dried in supercritical CO_2_.

**AlaNH_10_SIL:** The synthesis was performed analogous to SerNH_10_SIL.

**3,5-Bis(triisopropoxysilyl)aniline:** The sol–gel precursor was prepared and characterized as described in reference [49].

**ʟ-3,5-Bis(triisopropoxysilyl)aniline-Ser-NH_2_-Fmoc:** 0.972 g ʟ-Fmoc-Ser-OH (3.99 mmol) was dissolved in a mixture of 12 mL DMF and 12 mL DCM. The solution was cooled down to −12 °C and 2.97 mL *N*-methylmorpholine (1 M in DCM, 2.97 mmol), followed by 2.97 mL *n*-butylchloroformate (1 M in DCM, 2.97 mmol), was added. After 20 min, a solution of 1.49 g 3,5-bis(triisopropylsilyl)aniline in 15 mL DMF, 15 mL DCM, and 2.97 mL *N*-methylmorpholine (1 M in DCM) was added at −12 °C. The mixture was stirred overnight while it was warmed up to room temperature. The solvent was removed under vacuum. 20 mL pentane was added and non-soluble volatiles were removed by centrifugation. The resulting gel was purified by column chromatography in DCM/EE 10:1→6:1. Finally, 1.30 g ʟ-1,3-bis(triisoporpoxysilyl)aniline-Ser-NH-Fmoc (1.60 mmol, 54%) was obtained as a colorless gel.

^1^H NMR (400.1 MHz, toluol-*d*_8_) δ 1.27 (d, ^3^*J* = 6.16 Hz, 36H, iPr-CH_3_); 3.22 (s, 1H, Ser-OH); 3.41, 3.80 (m, 2 × 1H, Ser-CH_2_); 3.95 (t, ^3^*J* = 6.71 Hz, 1H, Fmoc-CH); 4.12 (ddd, 1H, Ser-CH); 4.27 (m, 2H, Fmoc-CH_2_); 4.40 (sept, ^3^*J* = 6.06 Hz, 6H, iPr-CH); 5.97 (d, *J* = 5.52 Hz, 1H, Ser-NH); 7.12 (dt separated by 1.24 Hz, ^3^*J* = 7.44 Hz, 2H), 7.18 (dt separated by 0.57 Hz, ^3^*J* = 7.42 Hz, 2H), 7.39 (d, ^3^*J* = 7.68 Hz, 1H), 7.42 (d, ^3^*J* = 7.46 Hz, 1H), 7.51 (d, ^3^*J* = 7.49 Hz, 2H) (all Fmoc-H_arom_); 8.13 (s, 1H, *p*-H_arom_); 8.34 (s, 2H, *o*-H_arom_); 8.83 (s, 1H, aniline-NH); ^13^C NMR (100.6 MHz, toluol-*d*_8_) δ 25.78 (iPr-CH_3_); 47.48 (Fmoc-CH); 56.32 (Ser-CH); 62.85 (Ser-CH_2_); 65.82 (iPr-CH); 67.51 (Fmoc-CH_2_); 120.14, 125.34, 127.37, 127.88 ppm (arom. Fmoc-CH); 128.22 (*o*-aniline-C); 133.96 (*m*-aniline-C); 137.57 (aniline C-N); 137.72 (*p*-aniline-C); 141.69, 141.73 (Fmoc-*tert*-C); 144.16, 144.26 (Fmoc-*tert*-C); 157.25 (Fmoc-C=O); 169.03 (Ser-C=O); ESI-MS (positive) *m*/*z*: main peaks at 849.36 (MK^+^), 833.39 (MNa^+^), 811.40 (MH^+^, calcd 811.39).

**ʟ-3,5-Bistri(isopropoxysilyl)aniline-Ala-NH_2_-Fmoc:** The precursor was prepared analogous to ʟ-3,5-bistri(isopropoxysilyl)aniline-Ser-NH_2_-Fmoc.

^1^H NMR (400.1 MHz, toluol-*d*_8_) δ 0.82 (Ala-NH_2_); 1.02 (d, ^3^*J* = 7.17 Hz, 3H, Ala-CH_3_); 1.28 (d, ^3^*J* = 6.12 Hz, 36 H, iPr-CH_3_); 3.99 (q, 1H, Ala-CH), 4,42 (sept., ^3^*J* = 6.12 Hz, 6H, iPr-CH), 7.74 (t, ^4^*J* = 0.95 Hz, 1H, *p*-H_arom_), 7.95 (d, ^4^*J* = 0.95 Hz, 2H, *o*-H_arom_), 9.28 (s, 1H, aniline-NH); ^13^C NMR (100.6 MHz, toluol-*d*_8_) δ 20.82 (Ala-CH_3_); 25.44 (iPr-CH_3_); 50.89 (Ala-CH); 65.40 (iPr-CH); 126.76 (*o*-aniline-C); 133.42 (*m*-aniline-C); 136.62 (*p*-aniline-C); 137.73 (aniline C-N); 172.26 (C=O).

**SerNH_10_oSIL:** 0.130 g ʟ-1,3-bis(triisopropoxysilyl)aniline-Ser-NH-Fmoc (0.161 mmol) was diluted in 0.384 g tetraisopropoxysilane (1.45 mmol) and 0.95 mL ethanol. 0.05 mL 1 M HCl was added under slight stirring. The solution was hydrolyzed at 60 °C for 4.5 h. After cooling to room temperature, 0.15 mL 1 M ammonia was added dropwise. For extraction of the monolithic aerogel, the material was overlaid with ethanol for 12 h. The supernatant was removed, and extraction was repeated for four times. Now a solution of 40% (v/v) of piperidine in toluene was overlaid at 40 °C until the material was completely transparent. Afterwards the supernatant was exchanged several times, first by toluene followed by acetone for supercritical drying.

**AlaNH_10_oSIL:** The material was prepared in analogy to SerNH_10_oSIL.

**(+)/(−)-3CP:** 3-Carboxy-2,2,5,5-tetramethylpyrrolidin-1-oxyl (3-carboxy-PROXYL or 3CP) was prepared according to [64]. The racemate separation was carried out with1-phenylethylamine [65]: 0.195 g 3CP (1.05 mmol) is dissolved in 20 mL acetone, and 0.134 mL (*S*)-(−)-1-phenylethylamine (1.05 mmol) is added, followed by crystallization. The precipitate is filtered off and washed with acetone. (The acetone phase can be used to obtain (−)-3CP). The filtrate is dissolved in water, acidified with hydrochloric acid to pH 3 and extracted three times with diethyl ether. The ether phase is dried with MgSO_4_, and the solvent is removed. A yellow solid is obtained, which becomes lighter with each subsequent separation step. The purity is checked at the specific rotation value of 79° in ethanol, and the procedure is repeated if necessary. After three separation steps, an enantiomeric excess of ee = 97% was achieved.

The procedure for obtaining (−)-3CP is identical to the procedure for (+)-3CP, except that (*R*)-(+)-1-phenylethylamine is used for separation. After three separation steps, an enantiomeric excess of ee = 95% was achieved.

**SH-FmocAlaNH*_z_*oSIL:** 3,5-bis(triisopropoxysilyl)aniline and 3,5-bis(triisopropoxysilyl)thiophenol are dissolved in ethanol (0.95 mL). Hydrochloric acid (0.05 mL, 1 M, 0.05 mmol) is added and the solution is stirred for 5 h. After hydrolysis, ammonia (0.15 mL, 1 M, 0.05 mmol) is added under vigorous stirring. The solution is stirred for 1 min and then transferred into a syringe. After 15 min, the solution becomes turbid. The material is aged for 1 d inside the sealed syringe.

**SH-AlaNH*_z_*oSIL:** SH-FmocAlaNH*_z_*oSIL was washed four times with ethanol, by replacing the solvent after 0.5 d. To cleave off the Fmoc group, the material was overlaid with 40% (v/v) piperidin in toluol for 3 d and then washed with toluol for 3 d.

**Ar_F_SH-FmocAlaNH*_z_*oSIL:** SH-FmocAlaNH*_z_*oSIL is added to a degassed solution of pentafluorostyrene (0.312 g, 1.61 mmoL) and DMPA (5 mg, 0.04 mmol) in 10 mL of toluol. The material was infiltrated overnight. The material in solution was irradiated with UV light (λ = 365 nm) for 6 h. The material was washed four times with toluol, by replacing the solvent after 0.5 d. Supercritical drying delivers the aerogel.

**TEMPONH*_x_*SIL:** A solution of 4-carboxy-TEMPO (100 mg/100 mg/100 mg/150 mg/200 mg) and pentafluorophenol (92 mg/92 mg/92 mg/138 mg/184 mg) is prepared in 10 mL ethyl acetate and cooled to 2 °C. One adds dicyclohexylcarbodiimide (DCC) (103.1 mg/103.1 mg/103.1 mg/154.3 mg/206.3 mg) and the system is heated to room temperature and stirred for 3 h. One adds 0.5 g NH*_x_*SIL (*x* = 1%/2%/5%/10%/15%) and stirs for a further 60 h at room temperature. The supernatant solution is removed and extracted for one day in 10 mL ethyl acetate. The supernatant solution is again removed and the material can be dried by supercritical CO_2_.

**TEMPONH*_y_*oSIL:** The material is prepared in analogy to TEMPONH*_x_*SIL. NH*_y_*oSIL is used as a starting material.

### Analyses

ATR-IR spectroscopy was performed on a Bruker Tensor with an ATR unit. All spectra were background-corrected and normalized to the Si–O–Si vibration. N_2_ physisorption measurements were performed with a Micromeritics Tristar 3020 at −196 °C. Prior to analysis, the samples were degassed at 60 °C for 12 h. Solid-state NMR spectra were recorded using a Bruker AVANCE III spectrometer operating at 400 MHz equipped with a 4 mm PH MAS DVT 400W1 BL4 N-P/H CGR probe head with magic angle gradient. ^1^H NMR measurements were performed on a Bruker Ascend 400 MHz spectrometer. Scanning electron microscopy and energy-dispersive X-ray spectroscopy were performed on a Hitachi Regulus SU8200. UV–vis spectroscopy was performed on an Agilent UV–Vis Cary 4000. Supercritical drying was carried out using a SPI-DRY critical point dryer Jumbo. HPLC measurements were performed on a Hewlett Packard 1100 Series.

**ESR spectroscopy:** ESR spectroscopy was performed on a Bruker Magnetech MS 5000 equipped with a variable temperature unit (TC-H03 Temperature Controller, magnetech GmbH). Prior to use, all materials and solutions were degassed under argon by at least 10 pump–freeze–thaw cycles. For each measurement, 50 mg of the representative material was infiltrated under argon overnight with 2 mL of a 2 mM solution of (+)-3CP or (−)-3CP in ethanol. Subsequently, the supernatant was removed, and the material was washed three times with pure degassed ethanol. At every temperature, the samples were allowed to equilibrate for at least 10 min prior to measuring the spectra. All spectra have been simulated using the free MATLAB toolbox easyspin [71] with two components of different rotational correlation time τ_c_. Spectra of (+)-3CP and (−)-3CP within the same material have been simulated in parallel using identical parameters when possible, only with adapted fractions of the two components of different τ_c_.

## Supporting Information

File 1 Materials characterization and ESR spectra.

## Data Availability

All data that supports the findings of this study is available in the published article and/or the supporting information of this article.
