# Supplementary material for: Stereodiscrimination of guests in chiral organosilica aerogels studied by ESR spectroscopy
Source: Beilstein J Nanotechnol. 2025 Nov 13;16:2034–54. doi: 10.3762/bjnano.16.140 (PMC12621621; doi:10.3762/bjnano.16.140)
Supplement: File 1 — Materials characterization and ESR spectra. [file Beilstein_J_Nanotechnol-16-2034-s001.pdf]

BEILSTEIN JOURNAL OF NANOTECHNOLOGY

Supporting Information

for

Stereodiscrimination of guests in chiral organosilica aerogels studied by ESR spectroscopy

Sebastian Polarz, Yasar Krysiak, Martin Wessig and Florian Kuhlmann

Beilstein J. Nanotechnol. **2025,** *16,* 2034–2054. doi:10.3762/bjnano.16.140

Materials characterization and ESR spectra

License and Terms: This is a supporting information file under the terms of the Creative Commons Attribution License (https://creativecommons.org/licenses/by/4.0). Please note that the reuse, redistribution and reproduction in particular requires that the author(s) and source are credited and that individual graphics may be subject to special legal provisions.
The license is subject to the *Beilstein Journal of Nanotechnology* terms and conditions: (https://www.beilstein-journals.org/bjnano/terms)

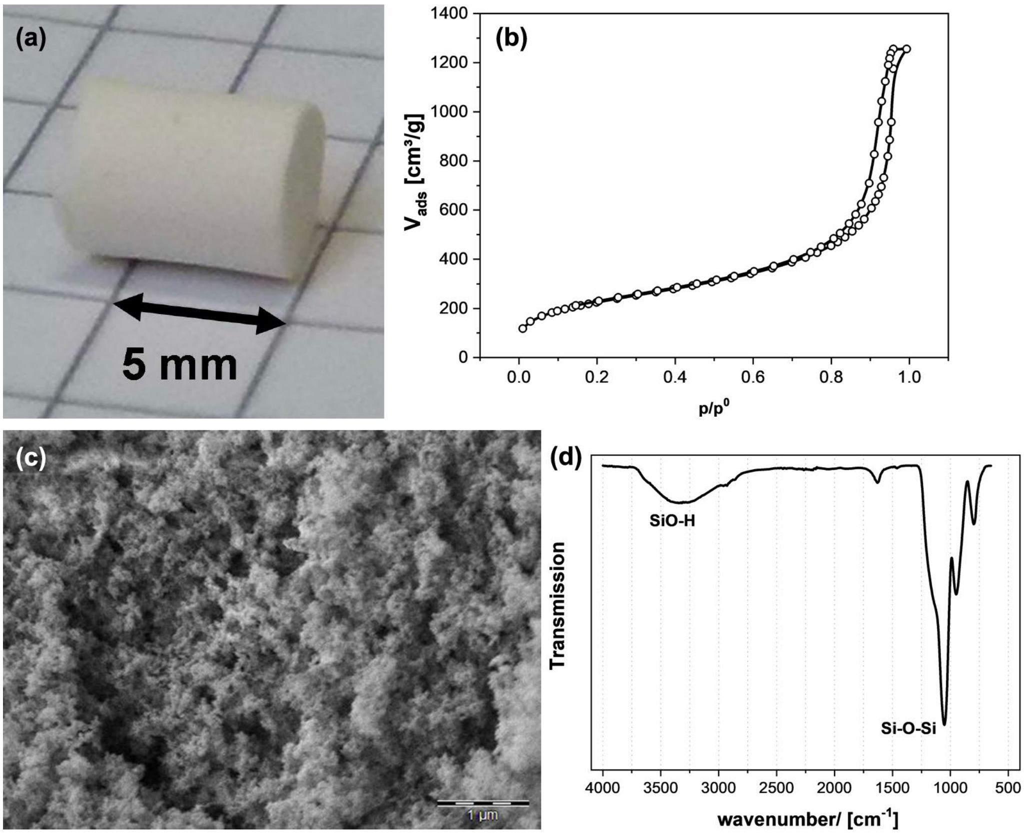

Figure S1: SIL-AG as achiral reference material. (a) Photographic image of silica monolith after supercritical drying. (b) Porosity analysis using N_2 physisorption measurements (A_{BET} = 946 $m^2 \cdot g^{-1}$). (c) SEM micrograph. (d) FTIR spectrum.

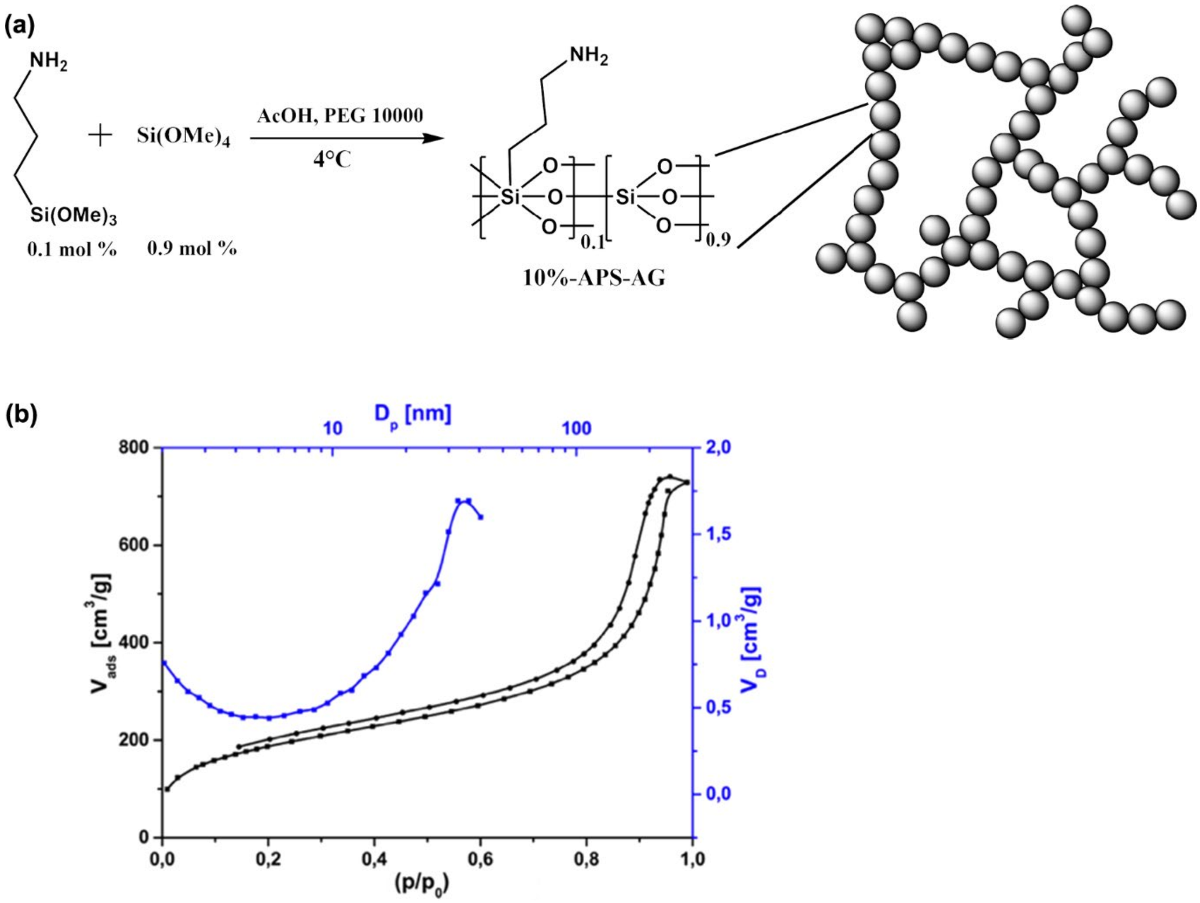

Figure S2: NH_xSIL. (a) Synthesis scheme. (b) Nitrogen physisorption analysis (black, A_{BET} = 681 $m^2 \cdot g^{-1}$) and pore-size distribution (blue).

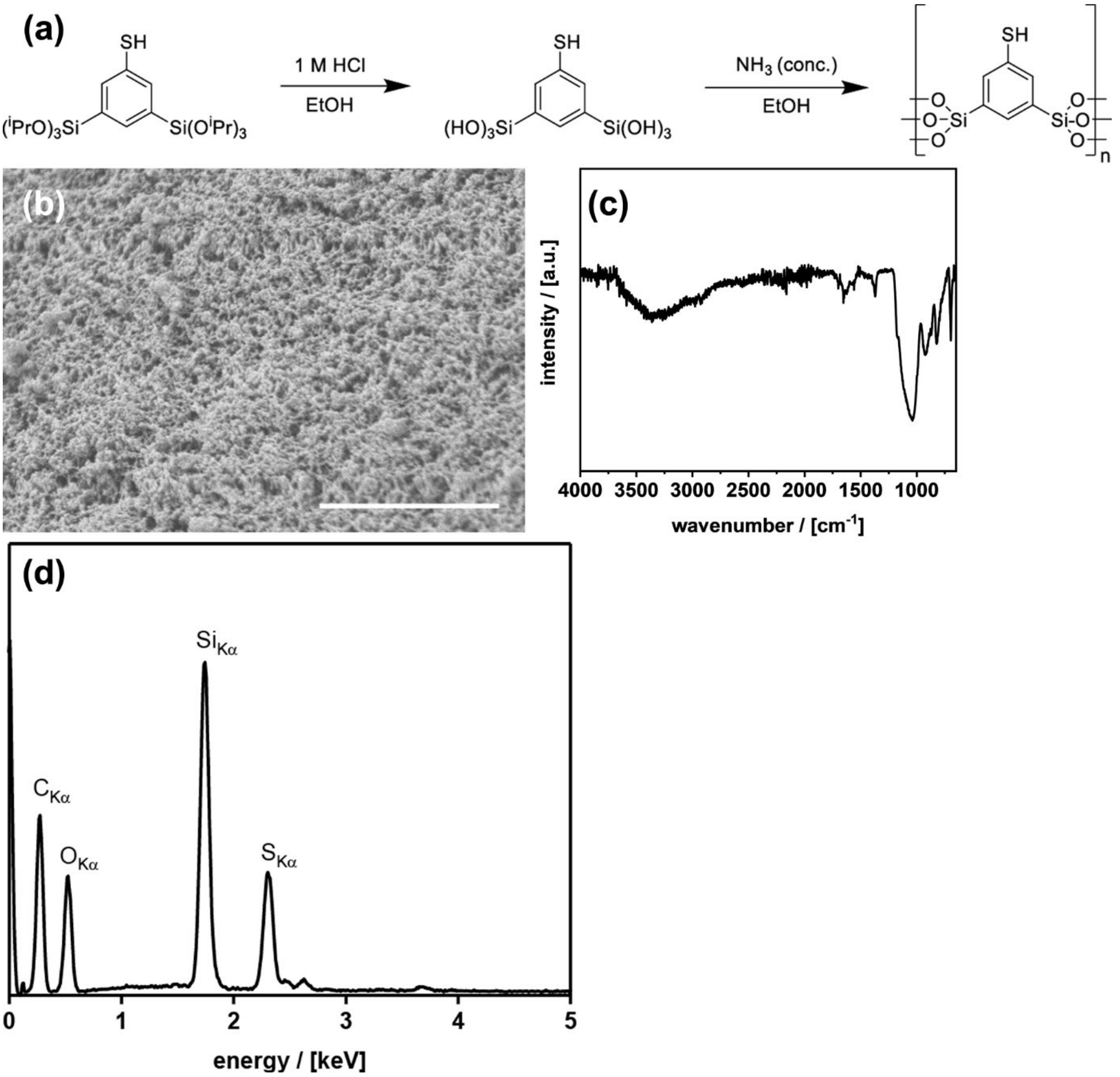

Figure S3: SH-AG as achiral reference material. (a) Sol–gel preparation. (b) SEM micrograph (scale bar = 2 µm). (c) FTIR spectrum (ν [cm^{-1}] = 3000–3200 (CH-arom), 1100 (Si–O–Si), 600–700 (CS deformation). (d) EDX spectrum.

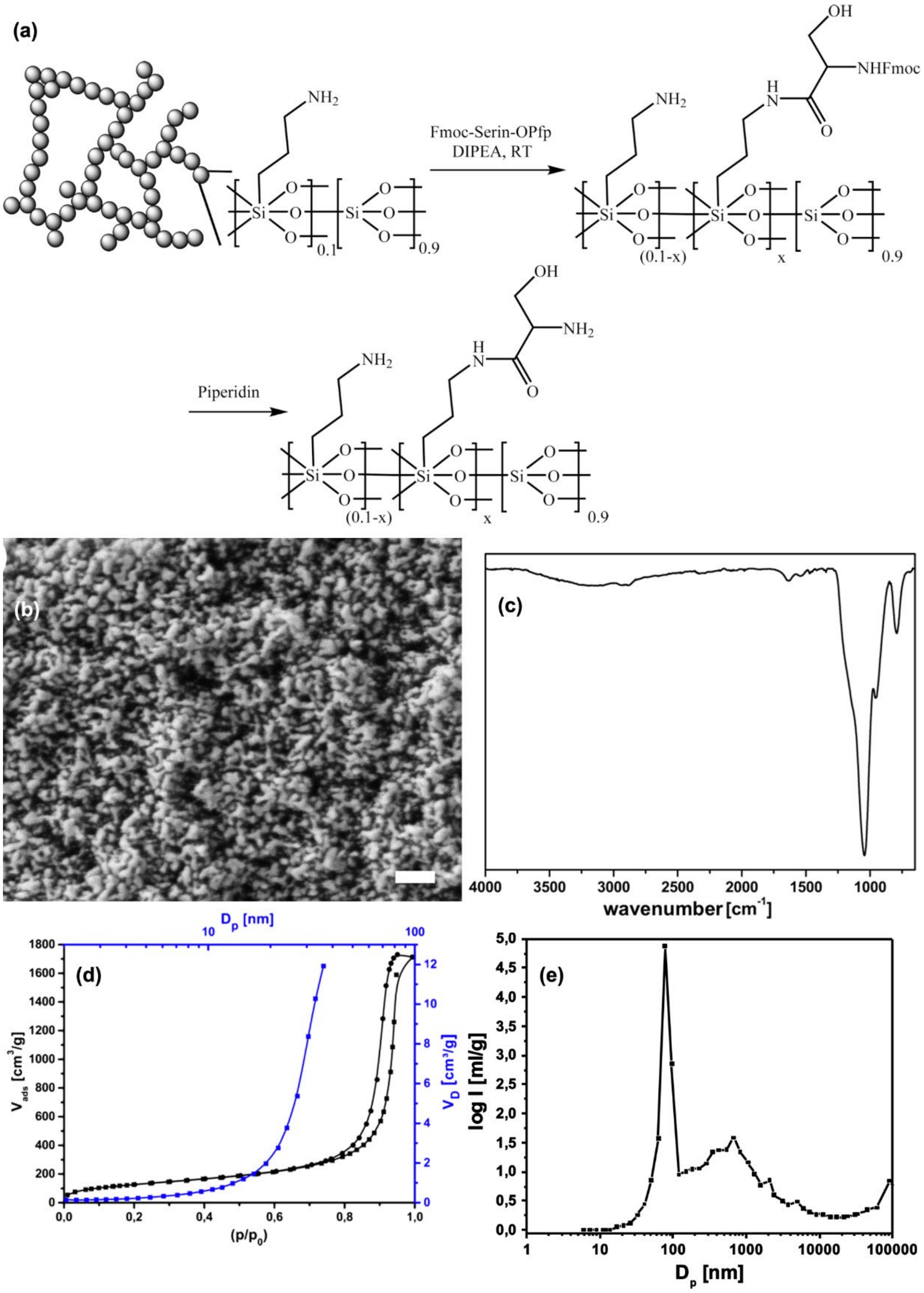

Figure S4: SerNH$_{10}$SIL. (a) Synthesis scheme. (b) SEM micrograph; scale bar = 200 nm. (c) Nitrogen physisorption analysis (black) (A_{BET} = 472 m^2·g^{-1}) and pore-size distribution (blue). (d) FTIR (ATR, v = 3200 cm^{-1} (OH), 2860–2970 cm^{-1} (CH), 1637 and 1541 cm^{-1} (amid I & II), 1049 cm^{-1} (SiO)). (e) Mercury intrusion porosimetry.

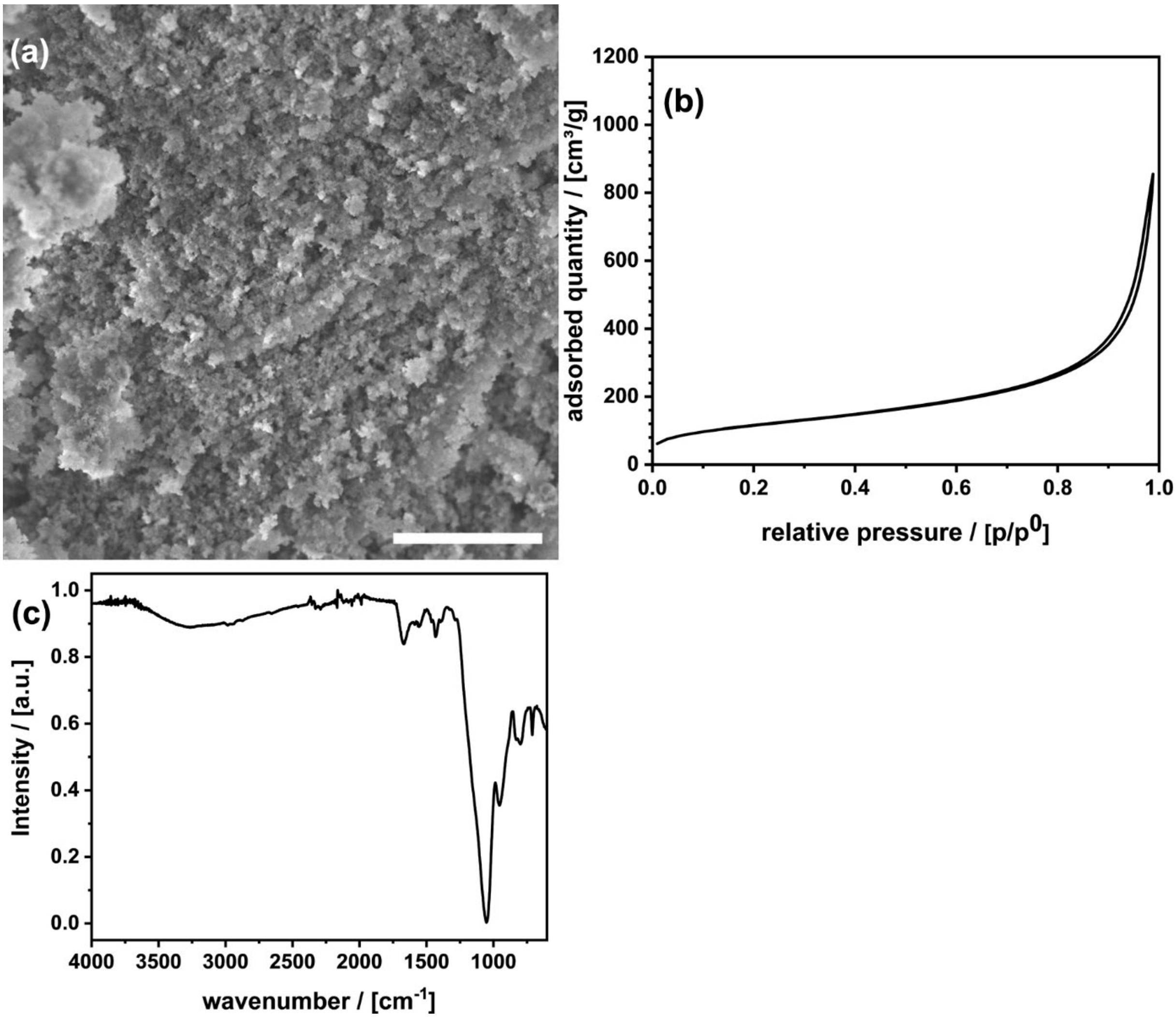

Figure S5: AlaNH15oSIL. (a) SEM micrograph; scale bar = 2 μm. (b) Nitrogen physisorption isotherm (A_{BET} = 404 $m^2 \cdot g^{-1}$). (c) FTIR (ATR, ν = 3400 cm^{-1} (NH), 3200 cm^{-1} (OH), 2978 & 2858 cm^{-1} (CH), 1668 cm^{-1} (amid I), 1548 cm^{-1} (amid II), 1368–1577 cm^{-1} (aliph. + arom. C–C); 1044 cm^{-1} (SiO).

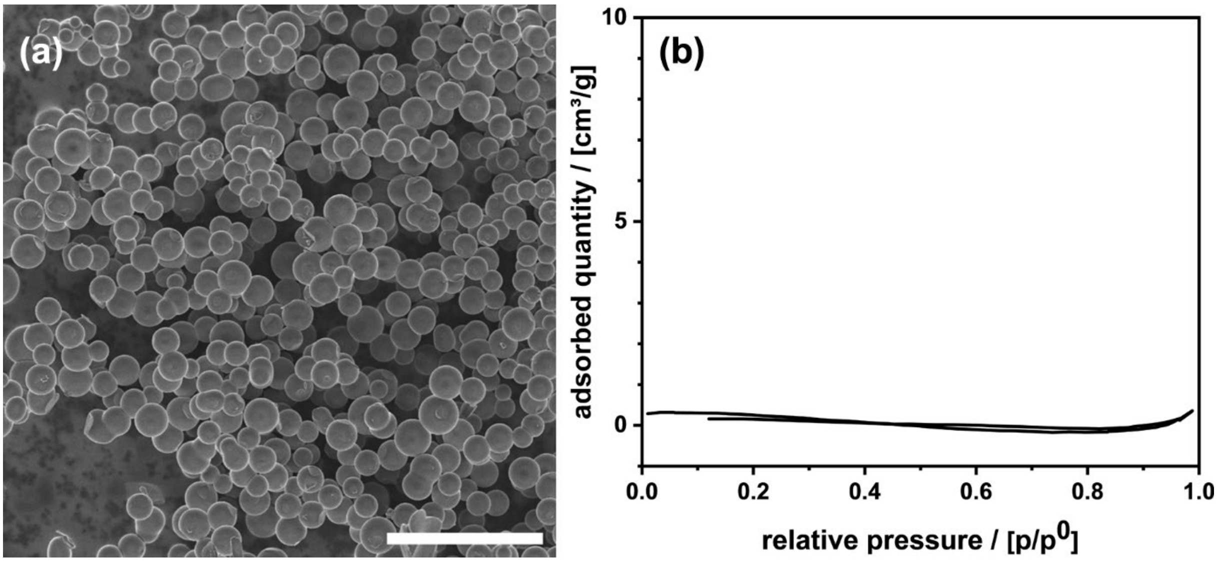

Figure S6: AlaNH15oSIL. (a) SEM micrograph; scale bar = 20 µm. (b) Nitrogen physisorption isotherm.

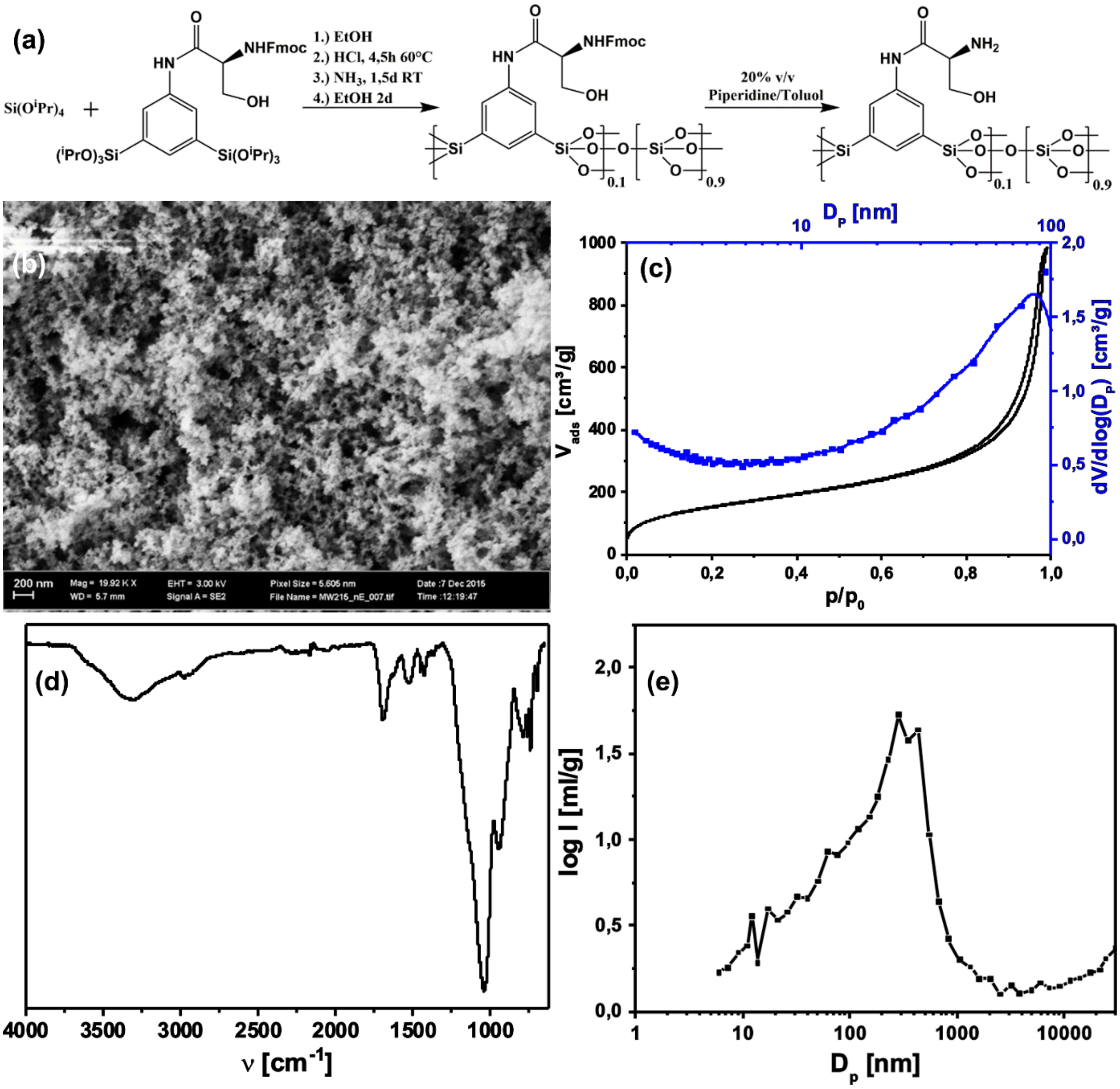

Figure S7: SerNH10oSIL. (a) Synthesis scheme. (b) SEM micrograph; scale bar = 200 nm. (c) Nitrogen physisorption analysis (black, A_{BET} = 552 $m^2 \cdot g^{-1}$) and pore-size distribution (blue). (d) FTIR spectroscopy (ATR, ν = 3000–3500 cm^{-1} (NH & OH), 2985 & 2945 & 2874 cm^{-1} (CH), 1673 cm^{-1} (arom. amid I), 1582 cm^{-1} (arom. amid II), 1423 cm^{-1} (amine), 1047 & 950 cm^{-1} (SiO)). (e) Mercury intrusion porosimetry.

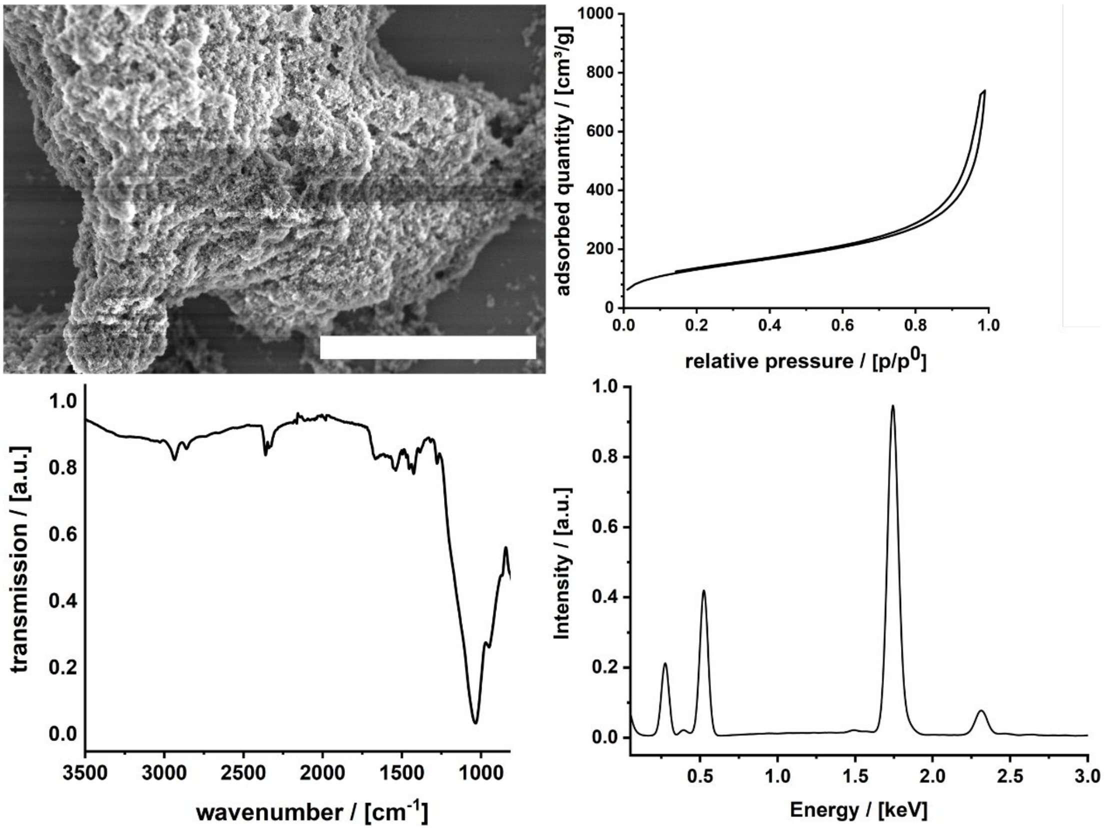

Figure S8: SH-AlaNH10oSIL. (a) SEM micrograph; scale bar = 5 µm. (b) Nitrogen physisorption analysis (black, A_{BET} = 478 $m^2 \cdot g^{-1}$). (c) FTIR spectroscopy (ATR, v = 2937 & 2858 cm^{-1} (CH), 1666 cm^{-1} (amid I), 1548 cm^{-1} (amid II), 1368–1466 (aliph. + arom. C–C); 1033 cm^{-1} (SiO)). (d) EDX analysis (0.28 eV (C), 0.4 eV (N), 0.52 eV (O), 1.43 eV (Al), 1.74 eV (Si), 2.32 eV (S)).

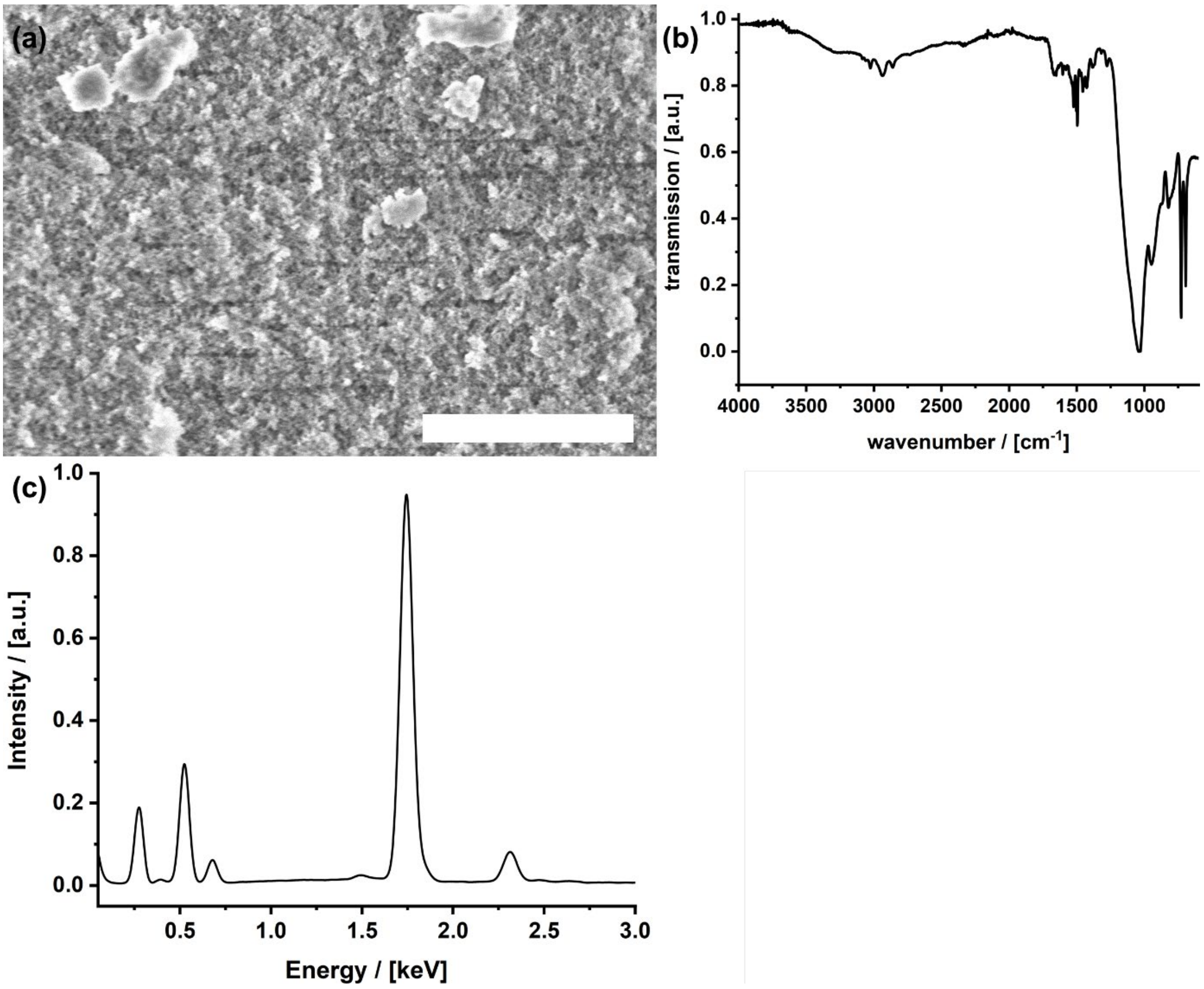

Figure S9: ArFSH-AlaNH10oSIL. (a) SEM micrograph; scale bar = 5 µm. (b) FTIR spectroscopy (ATR, ν = 3296 cm^{-1} (O–H), 2935 & 2860 cm^{-1} (CH), 1669 cm^{-1} (amid I), 1544 cm^{-1} (amid II), 1522 cm^{-1} (C–F), 1368–1449 (aliph. + arom. C–C); 1029 cm^{-1} (Si–O)). (c) EDX analysis.

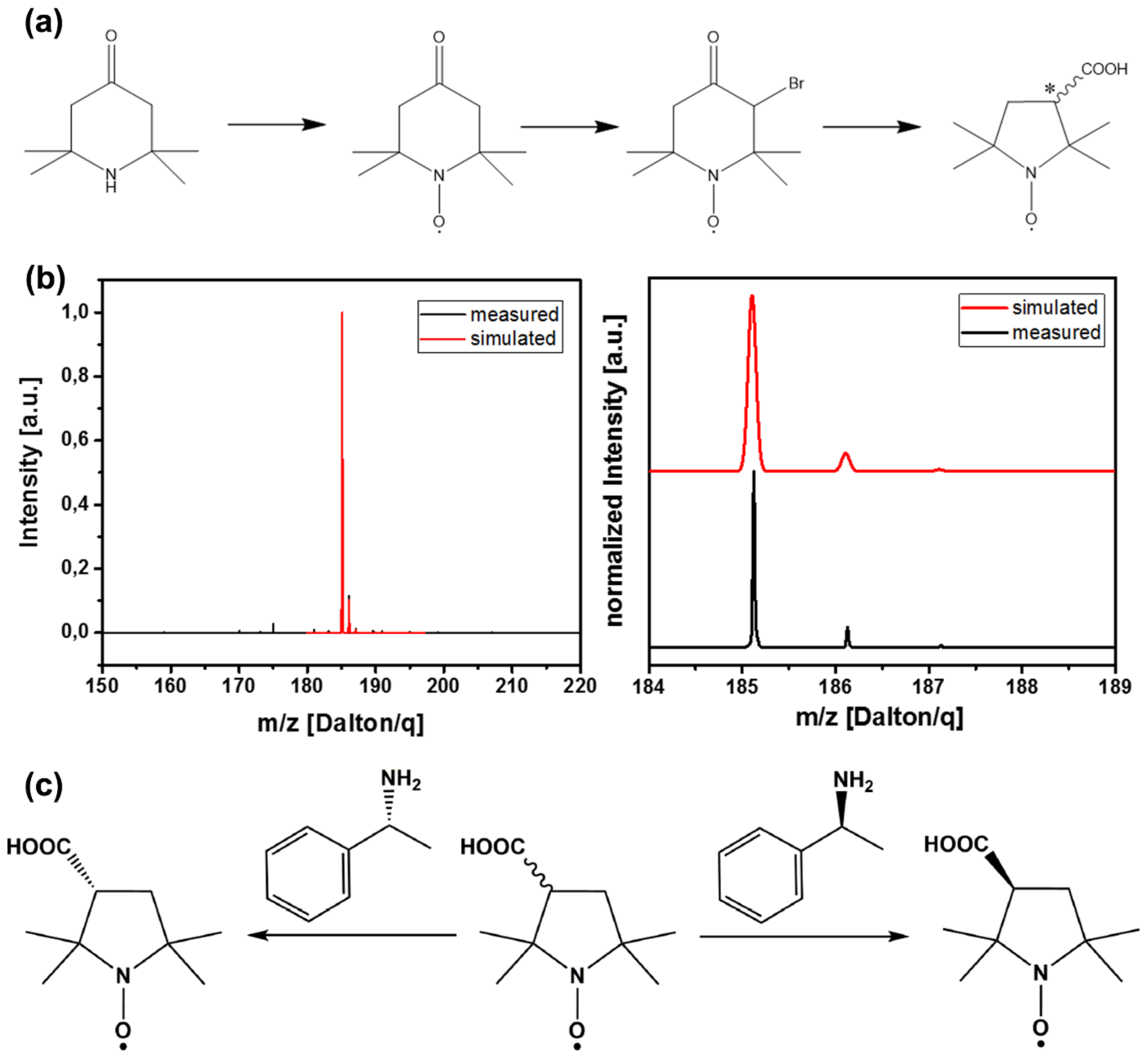

Figure S10: The 3CP spin probe. (a) Synthesis scheme. (b) Selected data for molecular characterization: ESI-MS. (c) Racemate separation of (+)-3CP.

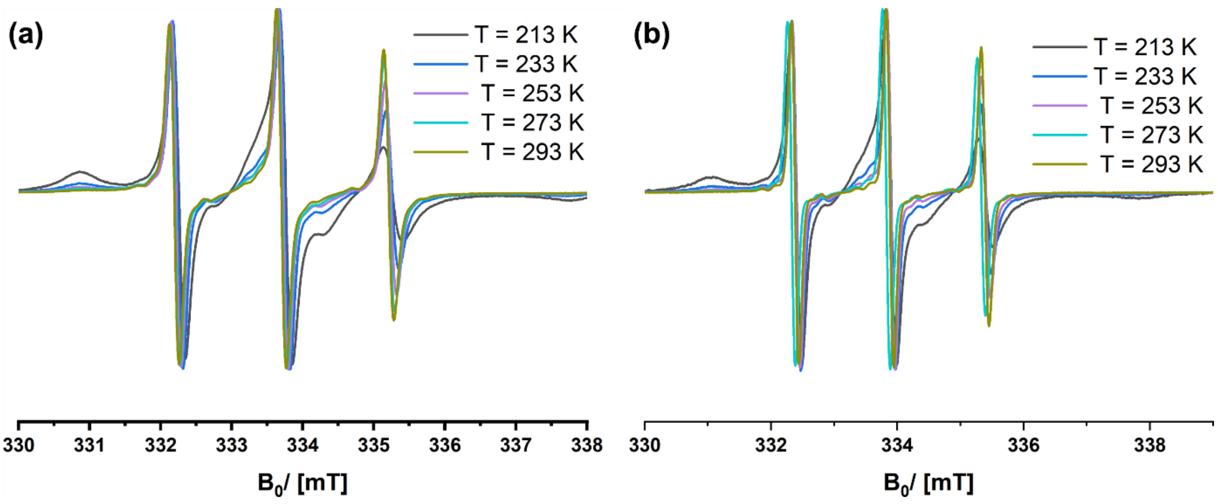

Figure 11: cw-ESR; SerNH10oSil; EtOH; temperature series. (a) (+)-3CP. (b) (−)-3CP.

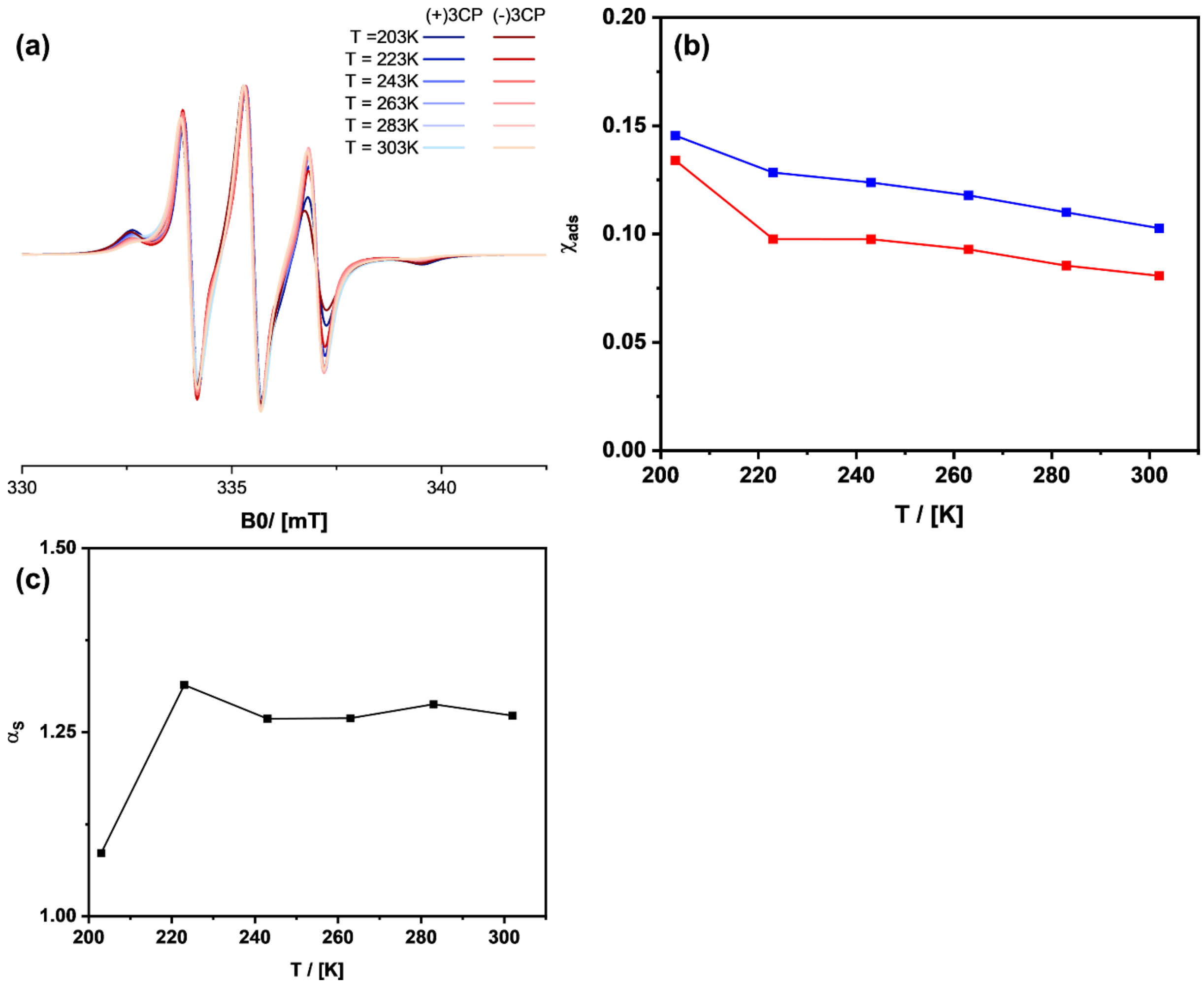

Figure 12: (a) cw-ESR; host material: AlaNH15oSIL; solvent ethanol/pentane 20:80. (b) Quantification of immobile species; (+)-3CP (blue), (−)-3CP (red). (c) Quantification of selectivity factor.

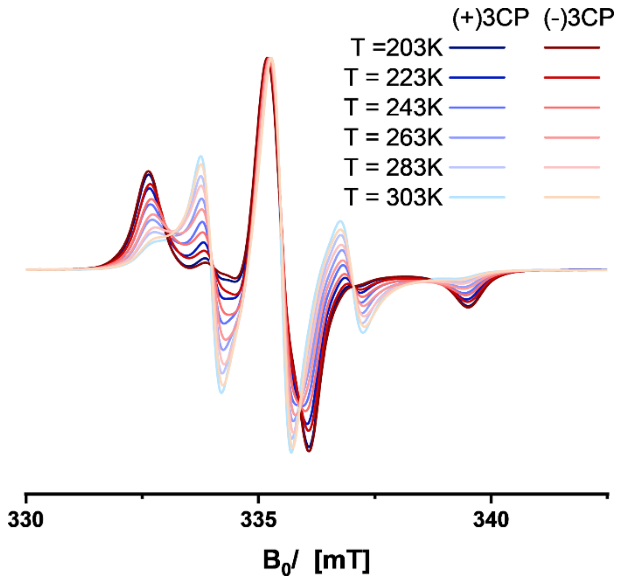

Figure S13: cw-ESR; host material: ArFSHAlaNH10oSIL; solvent: ethanol/pentane.
